# CCN1 Enhances Tumor Immunosuppression through Collagen‐Mediated Chemokine Secretion in Pancreatic Cancer

**DOI:** 10.1002/advs.202500589

**Published:** 2025-04-27

**Authors:** Hongjie Fan, Huzi Zhao, Lili Gao, Yucheng Dong, Pei Zhang, Pengfei Yu, Yunfei Ji, Zhe‐Sheng Chen, Xinmiao Liang, Yang Chen

**Affiliations:** ^1^ State Key Laboratory of Phytochemistry and Natural Medicines Dalian Institute of Chemical Physics Chinese Academy of Sciences Dalian 116023 China; ^2^ Ganjiang Chinese Medicine Innovation Center Nanchang 330000 China; ^3^ Department of Pathology Hubei Key Laboratory of Embryonic Stem Cell Research School of Basic Medical Science Hubei University of Medicine Shiyan 442000 China; ^4^ Department of Pathology Xinhua Hospital Affiliated to Medicine School of Shanghai Jiaotong University Shanghai 200082 China; ^5^ Peking Union Medical College Hospital Chinese Academy of Medical Sciences & Peking Union Medical College Beijing 100006 China; ^6^ Department of Mathematics University of Maryland College Park Maryland MD 20742 USA; ^7^ Department of Pharmaceutical Sciences College of Pharmacy and Health Sciences St. John's University Queens NY 11439 USA

**Keywords:** CCN1, collagen, immunosuppressive, pancreatic cancer, tumor microenvironment

## Abstract

Pancreatic ductal adenocarcinoma (PDAC) is characterized by a dense, immunosuppressive tumor microenvironment (TME) that limits therapeutic efficacy. This study investigates the role of cellular communication network factor 1 (CCN1, also known as Cyr61), an extracellular matrix‐associated protein, in modulating the TME of PDAC. It is demonstrated that Ccn1 promotes PDAC progression by upregulating collagen and chemokine expression, thereby facilitating immune cell exclusion and enhancing tumor growth. Using a Ccn1‐deficient PDAC model, decreased collagen and chemokine levels are observed, resulting in increased infiltration of cytotoxic immune cells and reduced myeloid‐derived suppressor cells (MDSCs). Furthermore, Ccn1‐deficient tumors exhibit heightened sensitivity to gemcitabine and show enhanced responsiveness to anti‐programmed cell death 1 (anti‐PD1) therapy. Mechanistically, Ccn1 regulates chemokine production through collagen expression, with chemokine levels remaining suppressed even upon interferon‐gamma treatment in collagen‐deficient cells. These findings highlight Ccn1 as a potential therapeutic target that reprograms the TME to enhance the efficacy of both chemotherapy and immunotherapy in PDAC, providing a novel approach for overcoming immune resistance in PDAC.

## Graphical abstract

CCN1 modulates the tumor microenvironment in pancreatic cancer by promoting collagen‐dependent chemokine production, thereby facilitating immune evasion. Deletion of CCN1 not only suppresses tumor growth but also enhances immune cell infiltration and increases tumor sensitivity to both chemotherapy and immunotherapy. These findings reveal a novel immunosuppressive pathway, highlighting CCN1 as a promising therapeutic target.

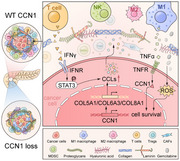

## 1 Introduction

Pancreatic ductal adenocarcinoma (PDAC) is one of the most aggressive solid malignancies, characterized by a high mortality rate,^[^ ^1^ ^]^ and is projected to be the second leading cause of cancer deaths by 2030.^[^ ^2^ ^]^ PDAC demonstrates remarkable resistance to chemotherapy, targeted therapies, and immunotherapy,^[^ ^3^, ^4^ ^]^ which is attributed to inherent intratumor heterogeneity and a highly desmoplastic and immunosuppressive tumor microenvironment (TME).^[^ ^5^, ^6^, ^7^ ^]^ PDAC is typically classified as an immunologically “cold” tumor. While checkpoint blockade immunotherapy has significantly improved outcomes in certain advanced cancers,^[^ ^8^ ^]^ its efficacy in PDAC remains limited.^[^ ^9^ ^]^ This resistance stems from the ability of PDAC to hijack stromal components to create a favorable TME that promotes tumor growth and impedes immunotherapy.^[^ ^6^ ^]^ The PDAC TME comprises cancer‐associated fibroblasts, immune cells, neurons, and an abundance of extracellular matrix (ECM) components, including collagen, fibronectin, and hyaluronic acid. The ECM contributes to increased intratumoral pressure, forming a physical barrier that hampers effective drug delivery and immune cell infiltration.^[^ ^10^ ^]^ Furthermore, the composition of TME undergoes dynamic changes throughout PDAC progression.

Hyaluronan, a key stromal ECM component in PDAC, is associated with poor prognosis. Human recombinant PH20 hyaluronidase has been shown to enhance drug delivery and improve chemotherapy efficacy, thereby inhibiting PDAC progression. Similarly, the Sonic hedgehog (SHH) signaling pathway, which is restricted to the stromal compartment, has been identified as a therapeutic target. Inhibition of SHH signaling enhances gemcitabine delivery efficiency in PDAC.^[^ ^11^ ^]^ Additionally, the secreted protein acidic and rich in cysteine (SPARC) overexpressed by fibroblasts in the PDAC TME, are positively associated with stroma.^[^ ^12^ ^]^ Targeting secreted protein acidic and rich in cysteine, particularly in combination with gemcitabine, has demonstrated the potential to prolong the overall survival of PDAC patients.^[^ ^13^ ^]^ Furthermore, Pin1 overexpression in cancer‐associated fibroblasts correlates with collagen deposition. Inhibition of Pin1 disrupts the immunosuppressive TME and enhances sensitivity to immunochemotherapy.^[^ ^14^ ^]^ Consequently, targeting the ECM has emerged as a critical component of therapeutic strategies for PDAC patients.

Cellular communication network factor 1 (CCN1), also known as cysteine‐rich angiogenic inducer 61 (CYR61), functions as an ECM‐associated factor and is a key member of the CCN family of matricellular proteins.^[^ ^15^ ^]^ Structurally, CCN proteins, including CCN1 through CCN6, consist of four distinct domains: an insulin‐like growth factor‐binding protein‐like domain I, a von Willebrand factor type C (VWC) repeat domain II, a thrombospondin type I repeat domain III, and a cysteine knot‐containing domain IV.^[^ ^16^ ^]^ CCN proteins also exhibit binding affinity towards various cell‐surface receptors, notably, several integrins.^[^ ^17^ ^]^ The VWC domain of CCN proteins interacts with members of the bone morphogenetic protein and transforming growth factor beta (TGFβ) family,^[^ ^18^ ^]^ while the carboxy‐terminal domain binds to fibronectin,^[^ ^19^ ^]^ vitronectin,^[^ ^18^ ^]^ and perlecan.^[^ ^20^, ^21^ ^]^

Functionally, CCN1 plays a critical role in promoting cell proliferation, tumor growth, and invasion across various malignancies. In breast cancer, CCN1 promotes tumor growth by enhancing vascular endothelial growth factor secretion through αvβ3 integrin receptors.^[^ ^22^ ^]^ Additionally, its interaction with urokinase‐type plasminogen activator receptor (uPAR) contributes to the progression of triple‐negative breast cancer.^[^ ^23^ ^]^ CCN1 overexpression correlates positively with β‐catenin activation in hepatocellular carcinoma and in osteoblasts.^[^ ^24^ ^]^ Moreover, Cyr61 facilitates gastric cancer cell migration and invasion via the αvβ3/NF‐κB‐dependent pathway.^[^ ^25^ ^]^ In pancreatic carcinogenesis, CCN1 promotes epithelial‐mesenchymal transition and stemness, or activates the Rac1/Akt/NF‐κB pathway.^[^ ^26^, ^27^ ^]^ Additionally, CCN1 suppresses the expression of deoxycytidine kinase (dCK), an enzyme crucial for gemcitabine activation, thereby conferring resistance to gemcitabine in pancreatic cancer cells.^[^ ^28^ ^]^ Notably, CCN1 also modulates immune cell migration, with inhibition of TYR03‐driven CCN1 secretion promoting macrophage polarization towards the M1 phenotype, enhancing the efficacy of anti‐programmed cell death 1 (anti‐PD1) therapy.^[^ ^29^, ^30^ ^]^ Given its multifaceted role, CCN1 emerges as a promising therapeutic target in pancreatic cancer.^[^ ^31^ ^]^

In our study, we observed that deletion of Ccn1 in pancreatic cancer cells results in enhanced infiltration of immune cells and suppression of tumor growth. Ccn1 regulates the expression of collagen, which modulates the expression of chemokines. Ccn1 depletion in pancreatic cancer cells promotes macrophage invasion and polarization towards the M1 phenotype. Moreover, Ccn1‐deficient pancreatic cancer cells exhibit elevated sensitivity to gemcitabine, with minimal activation of reactive oxygen species (ROS) level following treatment. Notably, combination therapy with gemcitabine and anti‐PD1 antibody yields enhanced efficacy in Ccn1‐deficient pancreatic cancer. Our findings elucidate the unrecognized role of Ccn1 in the TME and its mechanisms in regulating both immune cells and pancreatic cancer cells.

## 2 Results

### 2.1 CCN1 Expression is Positively Correlated with PDAC

**Figure 1:**
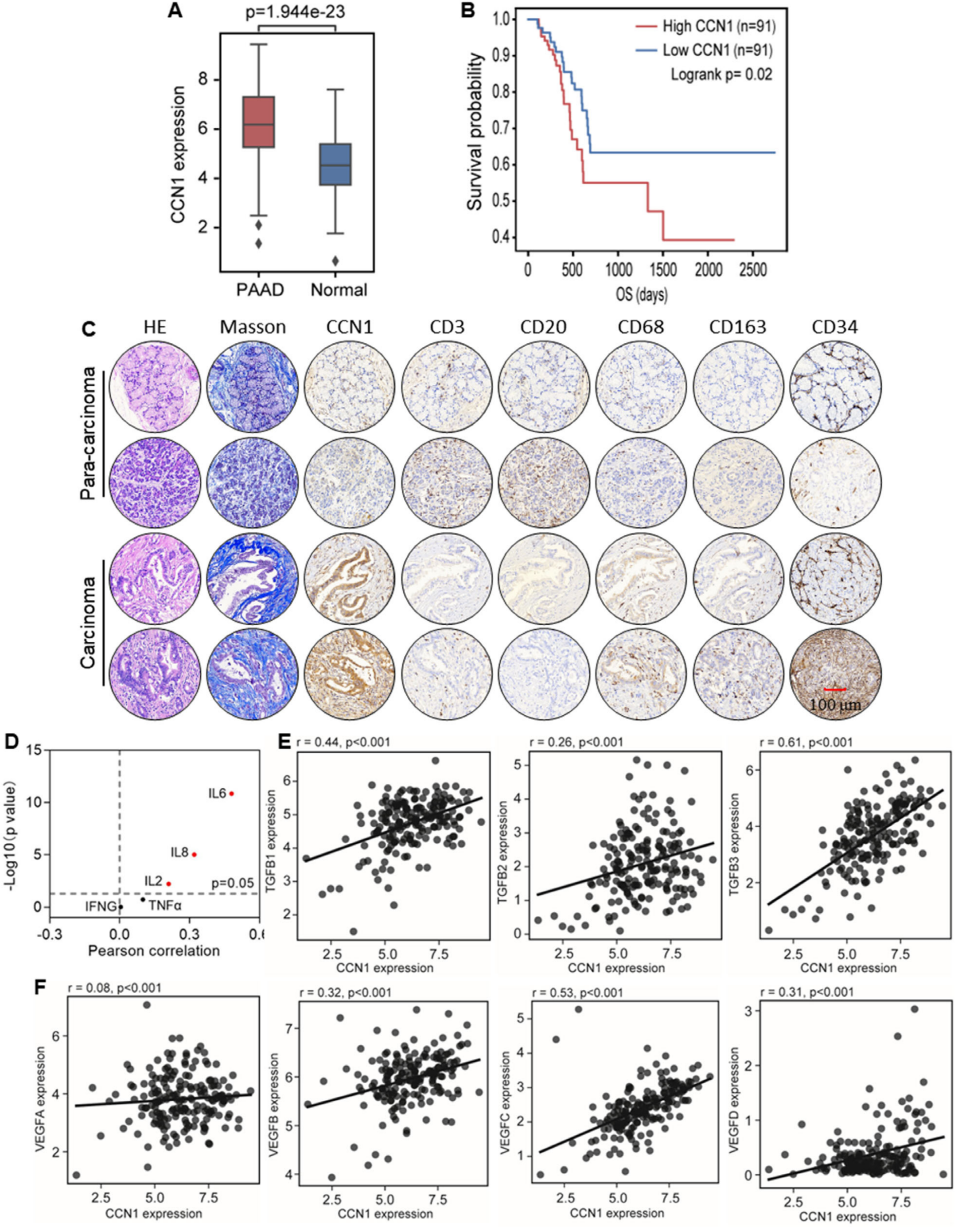
CCN1 is markedly upregulated in pancreatic cancer and associated with tumor progression. A) mRNA expression of CCN1 in human pancreatic cancer tissues (*n* = 182) and normal tissues (*n* = 165) were evaluated using the TCGA‐PAAD (pancreatic adenocarcinoma) and GTEx datasets. B) Kaplan–Meier analysis of overall survival in PAAD patients based on CCN1 expression. CCN1 high expression (*n* = 91), CCN low expression (*n* = 91). C) Representative images of H&E, Masson, and IHC staining comparing the protein expression of CCN1, CD3, CD20, CD68, CD163, and CD34 in human PDAC tissues and normal tissues. D) Spearman correlation analysis of mRNA levels between CCN1 and cytokines, including IL2, IL6, IL8, TNFα, and IFNG, evaluated using the TCGA‐PAAD dataset (*n* = 182). E) Spearman correlation analysis of mRNA levels between CCN1 and TGFB1, TGFB2, and TGFB3, evaluated using TCGA‐PAAD dataset (*n* = 182). F) Spearman correlation analysis of mRNA levels between CCN1 and VEGFA, VEGFB, VEGFC, and VEGFD, evaluated using the TCGA‐PAAD dataset (*n* = 182).

To investigate the role of CCN1 in PDAC, we analyzed CCN1 expressions in patients using The Cancer Genome Atlas (TCGA) database. CCN1 mRNA levels were significantly higher in PDAC tumor tissues than in normal pancreatic tissues, and high CCN1 expression was associated with shorter overall survival in patients (**Figure** **1**A,B). We further collected PDAC tissue samples to assess CCN1 expressions. Consistently, immunohistochemical and immunoblot results demonstrated that CCN1 expressions in PDAC were significantly higher than in adjacent normal tissues (Figure 1C; Figure S1A, Supporting Information). The results of Masson staining are consistent with previous reports indicating that abundant collagen forms a physical barrier, thereby promoting PDAC progression^[^ ^32^ ^]^ (Figure 1C). Interestingly, in CCN1‐expressing PDAC, macrophage infiltration and angiogenesis levels were notably higher compared to adjacent pancreatic tissues with lower CCN1 expression (Figure 1C; Figure S1B, Supporting Information). To further examine the relationship between CCN1 and immune modulation, we analyzed its correlation with various immune factors and found a strong positive association with Interleukin 6 (IL6), C‐X‐C motif chemokine ligand 8 (CXCL8), and tumor necrosis factor alpha (TNFα) (Figure 1D). CCN1 expression correlates with vascular endothelial growth factor A (VEGFA) levels, which is associated with immune cell infiltration in tumors, supporting our findings.^[^ ^33^ ^]^ Consistently, CCN1 mRNA levels were also positively correlated with VEGF and TGFβ (Figure 1E,F). These findings suggest the pivotal role of CCN1 in PDAC progression, particularly in modulating immune responses.

### 2.2 Enhanced Immune Cell Infiltration Inhibits Tumor Growth in Ccn1‐Deficient PDAC

**Figure 2:**
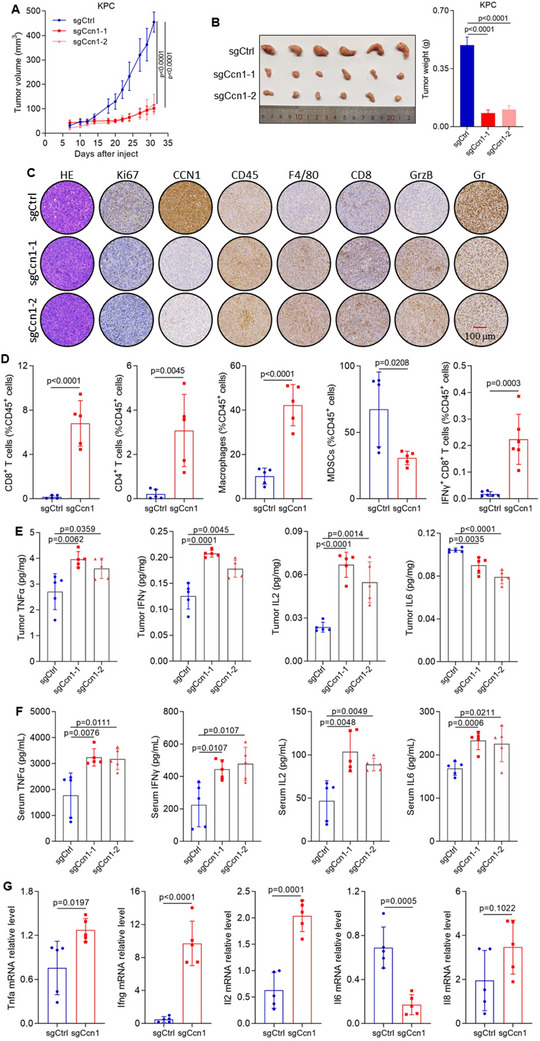
Ccn1 expression in PDAC correlates with immunosuppression. A) Tumor growth curves of sgCtrl and sgCcn1 KPC cells subcutaneously inoculated in C57B/L6 mouse. *n* = 6 mice per group. B) Representative images of tumors (left) and quantification of tumor weight (right) in sgCtrl and sgCcn1 KPC cell‐inoculated mice. C) Representative images of H&E, and immunohistochemistry analysis for Ki67, CCN1, CD45, F4/80, CD4, CD8, and Gr, markers in sgCtrl and sgCcn1 KPC subcutaneous tumors. D) Flow cytometric quantification of CD8^+^ T cells, CD4^+^ T cells, macrophages, MDSCs, and IFNγ^+^ CD8^+^ T cells in sgCtrl and sgCcn1 KPC tumors. E) Protein levels of TNFα, IFNγ, IL2, IL6 in sgCtrl and sgCcn1 KPC cells inoculated subcutaneous tumors. F) Protein levels of TNFα, IFNγ, IL2, IL6 in serum from mice bearing sgCtrl and sgCcn1 tumors. G) mRNA levels of Tnfa, Il2, Il6, and Il8 in sgCtrl and sgCcn1 KPC tumors.

To further elucidate the role of CCN1 in PDAC progression, we generated Ccn1 knockout in mouse pancreatic cancer cells KPC and Pan02 (Figure S2A,B, Supporting Information). We evaluated the cell proliferation of Ccn1 by subcutaneously injecting Ccn1 knockout KPC and Pan02 cells into nude mice and monitored the tumor volumes every two days until the mice were sacrificed for 25 days post‐implantation. Surprisingly, there was no significant difference in tumor volume between the Ccn1 knockdown group and the control group (Figure S2C,D, Supporting Information). To assess the antitumor efficacy of Ccn1 in PDAC cells, we injected KPC and Pan02 cells with Ccn1 knockout into immunocompetent mice. Notably, Ccn1‐KO significantly inhibited tumor growth in immunocompetent mice (**Figure** **2A,B**; Figure S2E,F, Supporting Information), while no significant weight loss was observed throughout the study (Figure S2G,H, Supporting Information), indicating that antitumor immunity contributed to the effects Ccn1 deficiency. These findings collectively suggest that Ccn1 depletion significantly inhibits PDAC tumorigenesis through immune modulation.

To further elucidate the role of Ccn1 in immune regulation, we analyzed immune cell subsets. Remarkably, Ccn1‐KO tumors exhibited a reduced proportion of MDSCs and an increased proportion of CD4^+^ T cells, CD8^+^ T cells, macrophages, and IFNγ^+^ CD8^+^ T cells (Figure 2C,D; Figure S3A, Supporting Information). Additionally, Ccn1‐KO tumors demonstrated significantly higher protein levels of TNFα, IL2, and IFNγ in tumor and serum (Figure 2E,F). Additionally, Ccn1‐KO tumors demonstrated significantly higher mRNA levels of Tnfa, Il2, and Ifng, while Il6 mRNA level decreased (Figure 2G). These results suggest an increase in cytotoxic immune cell infiltration in Ccn1‐deficient tumors, highlighting the essential role of Ccn1 in immune modulation. Interestingly, mRNA levels of Tgfb1, Vegfc, and Vegfd were significantly reduced in Ccn1‐deficient tumors, while mRNA levels of Vegfa and Vegfb remained unchanged (Figure S3B, Supporting Information). Further examination in Ccn1‐deficient KPC cells revealed decreased mRNA levels of Vdfa, Vegfb, Vegfd, and Tgfb in Ccn1‐deficient KPC cells (Figure S3C, Supporting Information).

### 2.3 CCN1 Deficiency Decreases Collagen and Chemokine Levels in PDAC

**Figure 3:**
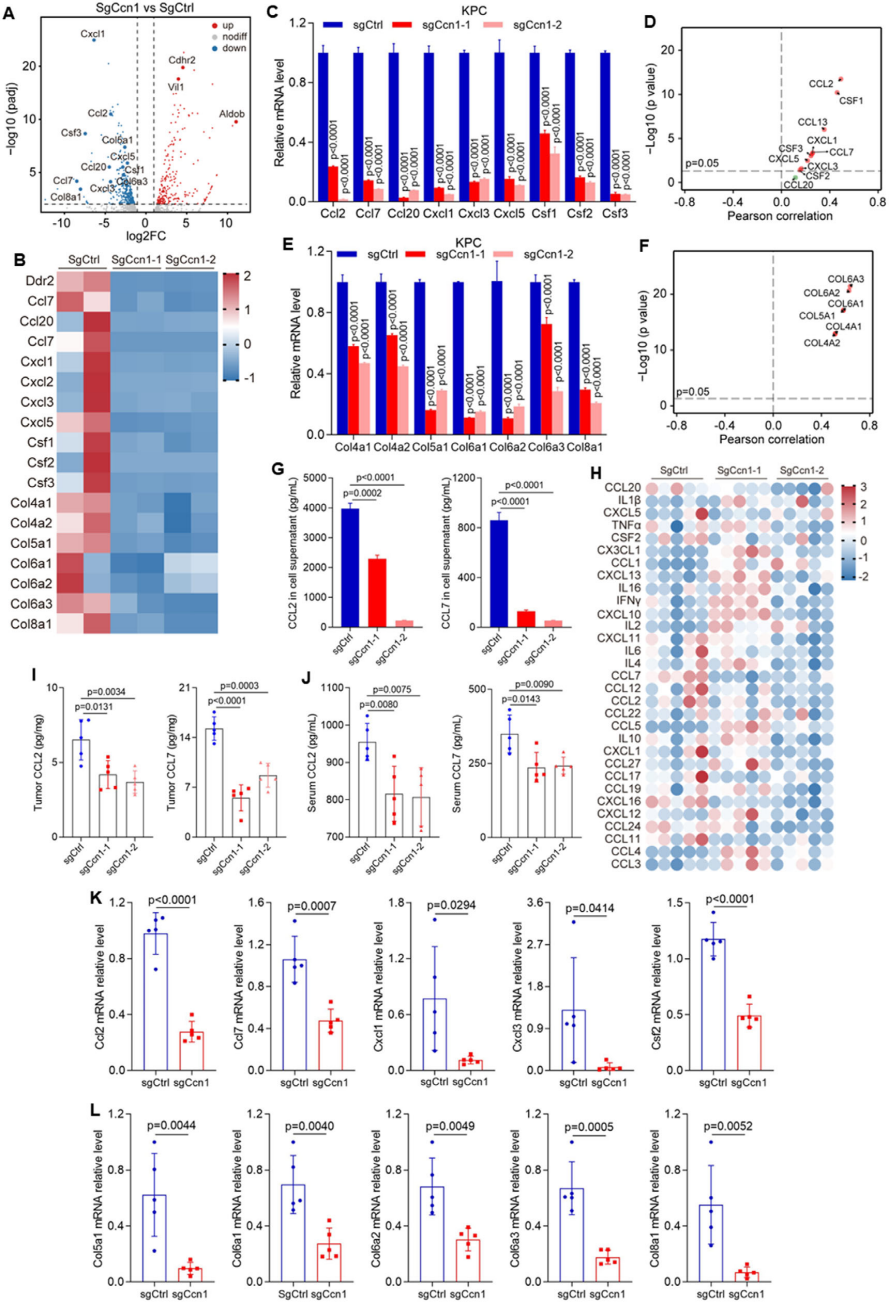
Ccn1 loss reduces the expression of chemokines and collagens in PDAC. A) Volcano plot illustrates the distribution of differential genes between sgCtrl and sgCcn1 KPC cells. B) Heatmap showing the mRNA expression of chemokines and collagens between sgCtrl and sgCcn1 KPC cells. C) mRNA levels of chemokines, including Ccl2, Ccl7, Ccl20, Cxcl1, Cxcl3, Cxcl5, Csf1, Csf2, and Csf3 between sgCtrl and sgCcn1 in KPC cells. D) Spearman correlation analysis of mRNA levels between CCN1 and chemokines based on the TCGA‐PAAD dataset (*n* = 182). E) mRNA levels of collagens, including Col4a1, Col4a2, Col5a1, Col6a1, Col6a2, Col6a3, and Col8a1 between sgCtrl and sgCcn1 in KPC cells. F) Spearman correlation analysis of mRNA levels between CCN1 and collagens based on the TCGA‐PAAD dataset (*n* = 182). G) Protein levels of CCL2 and CCL7 in cell supernatants from sgCtrl and sgCcn1 KPC cells. H) Heatmap showing the expression of a panel of cytokines in sgCtrl and sgCcn1 KPC tumors, as determined by Luminex multiplex assay. I) Protein levels of CCL2 and CCL7 in tumors from subcutaneous sgCtrl and sgCcn1 KPC tumors. J) Serum protein levels of CCL2 and CCL7 from mice bearing subcutaneous sgCtrl and sgCcn1 KPC tumors. K) mRNA expressions of Ccl2, Ccl7, Cxcl1, Cxcl3, and Csf2 in sgCtrl and sgCcn1 KPC tumors. L) mRNA expressions of Col5a1, Col6a1, Col6a2, Col6a3, and Col8a1 in sgCtrl and sgCcn1 KPC tumors.

To investigate the potential mechanisms by which CCN1 regulates the immune system and inhibits PDAC progression, we conducted RNA sequencing to analyze the gene expression profiles. Among the genes significantly downregulated in Ccn1‐KO KPC cells, there was a prominent decrease in collagens and chemokines (**Figure** **3**A). We illustrated the relative expression of key representative genes, showing consistent downregulation of chemokines such as Cxcl1, Cxcl3, and Ccl2, as well as collagens including Col5a1 and Col8a1, in Ccn1‐deficient KPC cells (Figure 3B). Significant decreases in mRNA levels of chemokines including Ccl2, Ccl7, Ccl20, Cxcl1, Cxcl3, Cxcl5, Csf1, Csf2, and Csf3, as well as collagens such as Col4a1, Col4a2, Col5a1, Col6a1, Col6a2, Col6a3, and Col8a1 were confirmed (Figure 3C–E), and the immunoblotting analysis also confirmed the reduction in COL5A1 protein levels (Figure S5A, Supporting Information), with consistent sequences observed in the TCGA‐pancreatic adenocarcinoma (PAAD) databases (Figure 3D,F). The secreted CCL2 and CCL7 were significantly decreased in Ccn1‐deficient KPC cells (Figure 3G). However, no significant reduction was observed in the protein levels of CCL20, CXCL1, CXCL5, CSF1, CSF2, or CSF3 (Figure S4A, Supporting Information).

We further analyzed the protein expression of chemokines in pancreatic tumor tissues. Protein levels of CCL2, CCL7, and CSF3 were significantly decreased in Ccn1‐deficient tumors (Figure 3H–I), whereas levels of CCL20, CXCL1, CXCL5, CSF1, and CSF2 remained unchanged (Figure S4B, Supporting Information). Consistently, CCL2 and CCL7 protein levels were also significantly reduced in the serum of Ccn1‐deficient mice (Figure 3J), while the levels of CCL20, CXCL1, CXCL5, CSF1, and CSF2 were unaffected (Figure S4C, Supporting Information). At the transcript level, mRNA expression of Ccl2, Ccl7, Cxcl1, Cxcl3, and Csf2 was markedly downregulated in Ccn1‐deficient pancreatic tumors (Figure 3K), whereas Cxcl5, Csf1, and Csf3 expression remained unchanged, and Ccl20 mRNA was elevated (Figure S5B, Supporting Information). Similarly, mRNA levels of Col5a1, Col6a1, Col6a2, Col6a3, and Col8a1 were significantly reduced in Ccn1‐deficient pancreatic tumors (Figure 3L), while Col4a1 and Col4a2 were unaffected (Figure S5C, Supporting Information). To the relationship between chemokines, collagens and immune infiltration, we analyzed a pancreatic cancer single‐cell RNA‐sequencing dataset containing 1 13 237 cells from 58 patients. This analysis revealed a negative correlation between chemokine/collagen expression and CD8^+^ T cell infiltration (Figure S4D, Supporting Information). In addition, the mRNA level of Cd274, which encodes programmed death‐ligand 1 (PD‐L1) protein, showed no significant changes (Figure S5D, Supporting Information). Collectively, these results reveal that Ccn1 promotes the expression of chemokines and collagens.

### 2.4 Collagens Modulate Chemokine Expression in PDAC Cells

**Figure 4:**
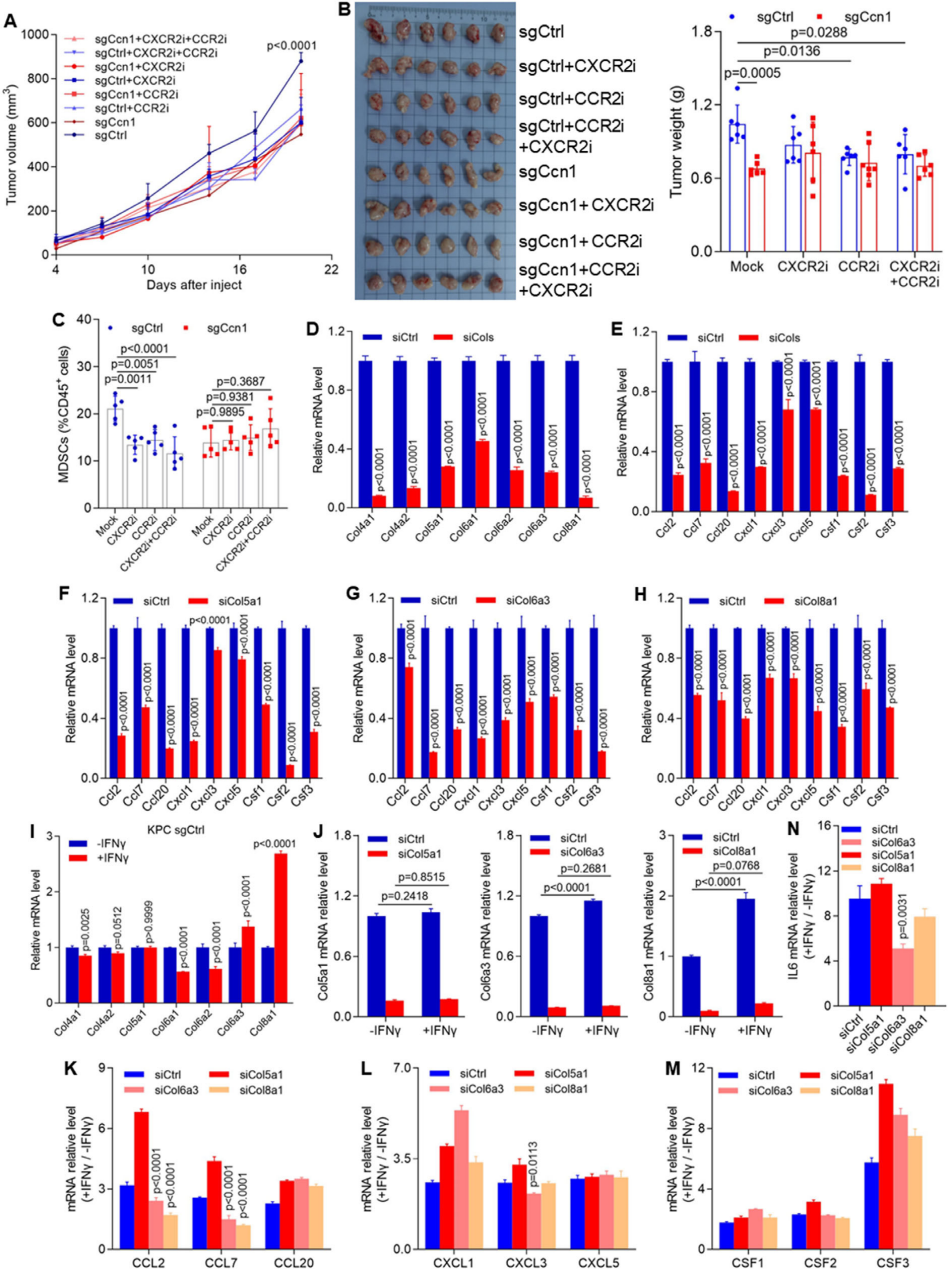
Collagens modulate chemokine expression in PDAC cells under IFNγ stimulation. A) Tumor growth of sgCtrl and sgCcn1 KPC cells inoculated subcutaneously in C57B/L6 mice. Tumor‐bearing mice were treated with CCR2 inhibitor RS504393 and CXCR2 inhibitor SB225002. *n* = 6 mice per group. B) Representative images of tumors (left) and quantification of tumor weight (right) in mice inoculated with sgCtrl or sgCcn1 KPC cells. Tumor‐bearing mice were treated with the CCR2 inhibitor RS504393 and the CXCR2 inhibitor SB225002. C) Flow cytometric analysis of MDSC proportions in subcutaneous sgCtrl and sgCcn1 KPC tumors. D) mRNA levels of collagens, including Col4a1, Cola4a2, Col5a1, Col6a1, Col6a2, Col6a3, and Col8a1, in KPC cells after their total knockdown. E) mRNA levels of chemokines, including Ccl2, Ccl20, Cxcl1, Cxcl3, Cxcl5, Csf1, Csf2, and Csf*3*, in KPC cells after their total knockdown. F–H) mRNA levels of chemokines, including Ccl2, Ccl7, Ccl20, Cxcl1, Cxcl3, Cxcl5, Csf1, Csf2, and Csf3, in KPC cells after Col5a1 knockdown (F), Col6a3 knockdown (G), and Col8a1 knockdown (H). I) mRNA levels of collagens, including Col4a1, Cola4a2, Col5a1, Col6a1, Col6a2, Col6a3, and Col8a1, in sgCtrl KPC cells after treatment with 100 ng mL^−1^ IFNγ. J) mRNA levels of Col5a1, Col6a3, and Col8a1 measured in siCol5a1, siCol6a3, and siCol8a1 KPC cells after treatment with 100 ng mL^−1^ IFNγ. K–M) mRNA levels of chemokines including the Ccls (K), Cxcls (L), and Csfs (M), in siCol5a1, siCol6a3, and siCol8a1 KPC cells after treatment with 100 ng mL^−1^ IFNγ. N) mRNA level of Il6 in siCol5a1, siCol6a3, and siCol8a1 KPC cells after treatment with 100 ng mL^−1^ IFNγ.

To confirm the inhibition of tumor growth associated with decreased chemokine expression in Ccn1‐KO tumor, we focused on CXC chemokines, including CXCL1, CXCL3, and CXCL5, which bind to CXCR2, as well as CCL2 and CCL7, which bind to CCR2. These chemokines regulate the infiltration and migration of various immune cells, such as monocytes, memory T lymphocytes, and NK cells, which play critical roles in the immune response.^[^ ^34^, ^35^ ^]^ Inoculation of Ccn1‐KO KPC and control cells into the subcutaneous flank of C57BL/6J mice, followed by treatment with SB225002 (a selective CXCR2 antagonist) and RS504393 (an antagonist of CCR2), revealed that tumor volume decreased significantly with CCR2 inhibitor or combined treatment in the control group pancreatic tumors (**Figure** **4**A,B). The proportion of MDSCs was decreased in Ccn1 tumors following treatment with a CCR2 inhibitor, a CXCR2 inhibitor, or their combination (Figure 4C). However, this effect was not observed in the Ccn1‐deficient pancreatic tumors, indicating that Ccn1 may promote tumor growth through the CCL‐CCR2 signaling pathways in PDAC.

To further explore the relationship between collagen and chemokines, we performed a combined knockdown of several collagen genes, including Col4a1, Col4a2, Col5a1, Col6a1, Col6a2, Col6a3, and Col8a1, which led to a significant reduction in chemokine expression of Ccl2, Ccl7, Ccl20, Cxcl1, Cxcl3, Cxcl5, Csf1, Csf2, and Csf3 in collagen‐deficient KPC cells (Figure 4D,E). To identify specific collagens responsible for chemokine regulation, we individually knocked down each collagen, observing a significant reduction in chemokine levels in each case (Figure 4F–H; Figure S6A–E, Supporting Information). Previous studies have shown that selective deletion of type I collagen (Col1) in pancreatic cancer myofibroblasts leads to the upregulation of Cxcl5 in cancer cells and suppression of CD8^+^ T cells.^[^ ^36^ ^]^ To investigate whether Ccn1 promotes chemokine expression through the regulation of collagen, we examined the effects of Col5a1 overexpression in Ccn1‐deficient KPC cells. Following transfection with Col5a1, mRNA levels of chemokines such as Cxcl1, Cxcl3, Cxcl5, Ccl20, Csf1, Csf2, and Csf3 were restored in Ccn1‐deficient KPC cells (Figure S7A–C, Supporting Information). However, mRNA expression levels of Ccl2 and Ccl7 remained unchanged (Figure S7A, Supporting Information). These findings suggest that Ccn1 promotes chemokine expression by upregulating collagen, thereby contributing to PDAC progression.

### 2.5 Ccn1 Deficiency Increases Susceptibility to TNFα‐Induced Cell Death and Leads to Unresponsiveness to IFNγ

IFNγ decreases collagen gene expression through transcription regulatory factor X5 (RFX5) and class II major histocompatibility complex transactivator (CIITA) in fibroblast cells.^[^ ^37^ ^]^ To further investigate the effect of IFNγ on collagen expression in our model, we treated KPC cells with IFNγ. The results showed a significant upregulation of Col6a3 and Col8a1 mRNA in KPC cells following IFNγ stimulation (Figure 4I). Surprisingly, this upregulation of Col6a3 and Col8a1 mRNA was also observed in Ccn1‐KO KPC cells after IFNγ treatment (Figure S8A,B, Supporting Information), indicating that collagen expression in response to IFNγ is independent of Ccn1 regulation. Additionally, IFNγ treatment increases CXCL9 and CXCL10 secretion in pancreatic tumor cells.^[^ ^38^ ^]^ To investigate whether collagen expression influences chemokine levels following IFNγ stimulation, we conducted collagen knockdown in KPC cells, followed by IFNγ treatment. In wild‐type KPC cells, IFNγ stimulation led to increased mRNA levels of Col6a3 and Col8a1, while Col5a1 levels remained unaffected. These findings suggest that IFNγ selectively upregulates certain collagen (Figure 4J), which may play a role in modulating chemokine expression in the TME. However, chemokine expression significantly increased with IFNγ treatment, and chemokine levels were lower in siCol6a3 cells compared to control KPC cells treated with IFNγ (Figure 4K–M). Interestingly, IL‐6 expression was higher in siCol6a3 cells than in control KPC cells following IFNγ stimulation (Figure 4N). These findings suggest that Ccn1 regulates chemokine expression through Col6a3 in KPC cells and highlights a complex mechanism by which Ccn1 enhances chemokine production to promote tumor progression.

**Figure 5:**
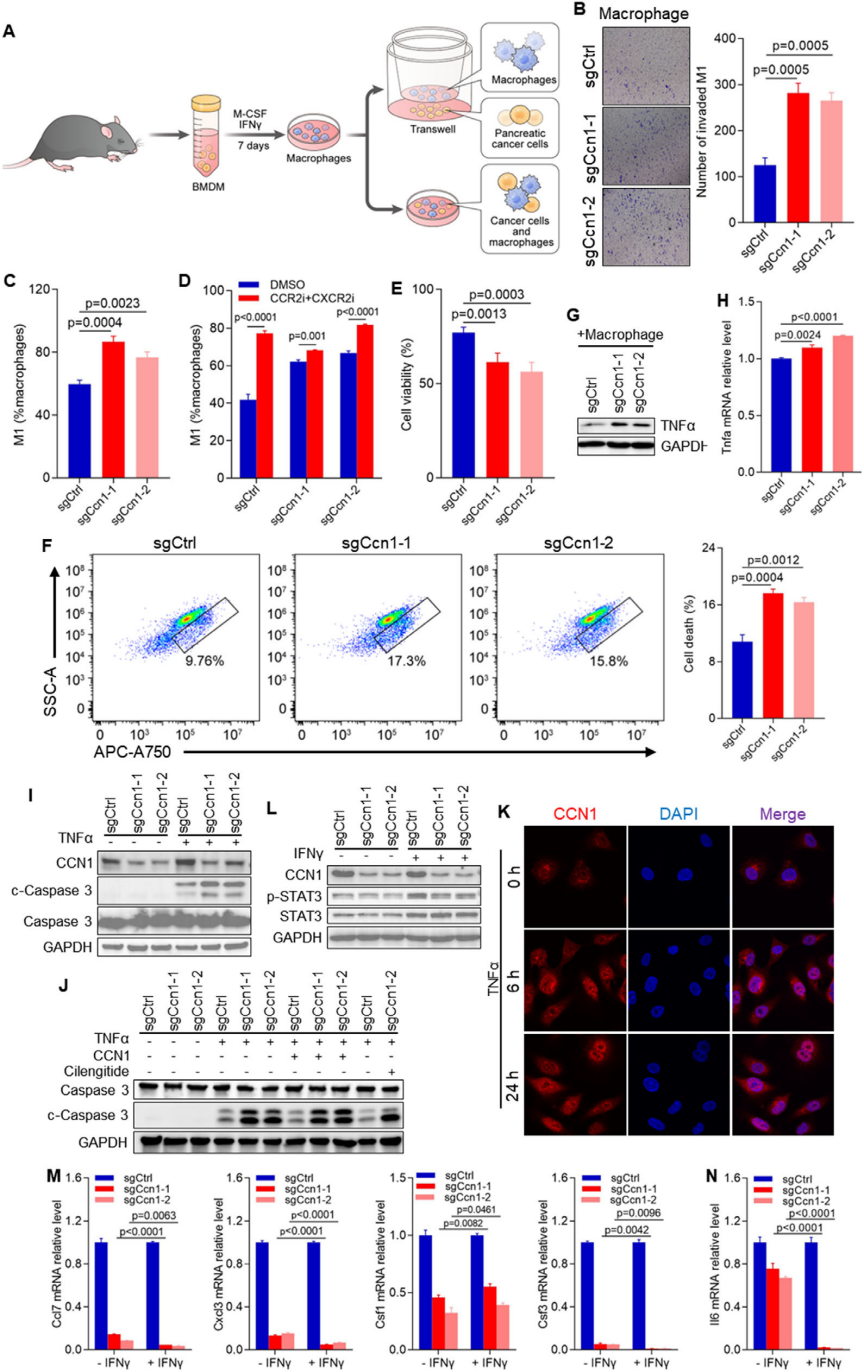
Ccn1 regulates the TME in PDAC. A) Schematic representation of macrophage isolation from mouse bone marrow. B) Macrophage invasion evaluated by the chemotactic effect of sgCtrl and sgCcn1 KPC cells in a Transwell invasion assay. C) Macrophage polarization assessed by flow cytometry in macrophages cocultured with sgCtrl and sgCcn1 KPC cells. D) Macrophage polarization assessed by flow cytometry in macrophages cocultured with sgCtrl and sgCcn1 KPC cells after treatment with CCR2 inhibitor RS504393 and CXCR2 inhibitor SB225002. E) Survival of sgCtrl and sgCcn1 KPC cells cocultured with macrophages, assessed by CCK‐8 assay. F) Cell death of sgCtrl and sgCcn1 KPC cells cocultured with macrophages, assessed by flow cytometry. G) Protein expression levels of TNFα in macrophages cocultured with sgCtrl and sgCcn1 KPC cells. H) mRNA expression levels of TNFα in macrophages cocultured with sgCtrl and sgCcn1 KPC cells. I) Protein expression level of c‐Caspase 3 in sgCtrl and sgCcn1 KPC cells after treatment with 100 ng mL^−1^ TNFα. J) Protein expression level of c‐Caspase 3 in sgCtrl and sgCcn1 KPC cells after treatment with 100 ng mL^−1^ TNFα, mouse CCN1 protein, and cilengitide (αVβ3 and αVβ5 integrin inhibitor). K) Nucleus localization of CCN1 in KPC cells with CCN1 (red) and DAPI (blue) fluorescence. L) Protein expression level of p‐STAT3 in sgCtrl and sgCcn1 KPC cells after treatment with 100 ng mL^−1^ INFγ. M) mRNA levels of Ccl7, Cxcl3, and Csf3 in sgCtrl and sgCcn1 KPC cells after treatment with 100 ng mL^−1^ INFγ. N) mRNA level of Il6 measured in sgCtrl and sgCcn1 KPC cells after treatment with 100 ng mL^−1^ INFγ.

CCL2 has been reported to play a critical role in macrophage recruitment and residence.^[^ ^39^ ^]^ We assessed the impact of Ccn1 deletion in KPC cells on macrophage recruitment by placing tumor cells in the Transwell chamber and immune cells above, with a layer of Matrigel on top (**Figure** **5**A), which demonstrated that Ccn1‐deficient KPC cells induced greater macrophages migration (Figure 5B). To investigate whether Ccn1‐deficient KPC cells affect macrophage polarization, we cocultured tumor cells and M1 macrophages and measured the proportion of M1 macrophages. The proportion of M1 macrophages cocultured with Ccn1‐deficient tumor cells was significantly higher (Figure 5C). To further evaluate whether Ccn1 promotes the polarization of M1 macrophages to M2 macrophages by augmenting chemokine expression, we cocultured KPC cells and M1 macrophages, treating them with RS504393 and SB225002. The results showed a significant increase in the proportion of M1 macrophages when treated with RS504393 and SB225002 (Figure 5D). We next analyzed tumor cell death during coculture with macrophages and found that Ccn1‐deficient cells exhibited increased susceptibility to macrophage‐induced cell death (Figure 5E,F). Additionally, both protein and mRNA levels of TNFα were elevated in macrophages co‐cultured with Ccn1‐deficient cells (Figure 5G,H). These findings suggest that Ccn1 not only limits macrophage recruitment but also promotes the polarization of macrophages from the M1 to the M2 phenotype.

When macrophages encounter tumor cells, they can directly engulf and kill tumor cells while also secreting TNFα to induce tumor cell death.^[^ ^40^, ^41^ ^]^ We aimed to investigate the role of Ccn1 in mediating TNFα resistance. We treated Ccn1‐KO KPC cells with TNFα and observed that Ccn1‐deficient KPC cells were more sensitive to TNFα‐induced cell death (Figure 5I). Similarly, Ccn1‐deficient Pan02 cells displayed heightened sensitivity to TNFα‐induced cell death (Figure S8C, Supporting Information). We hypothesized that CCN1 binds to integrins to activate downstream signaling pathways that inhibit TNFα‐induced killing. However, Ccn1‐deficient KPC cells remained sensitive to TNFα‐induced cell death even when treated with cilengitide or CCN1 protein (Figure 5J), suggesting that Ccn1 does not confer resistance to TNFα killing via integrin binding. We observed that CCN1 entered the nucleus upon TNFα treatment (Figure 5K), indicating that CCN1 protein enters the cell nucleus to confer resistance to TNFα‐induced killing. TNF binding to TNFR1 leads to NF‐κB activation, ultimately resulting in cell death.^[^ ^42^, ^43^ ^]^ However, the phosphorylation of NF‐κB by TNFα was unaffected in Ccn1‐deficient KPC cells (Figure S8D, Supporting Information).

In tumor cells, hyperactivated signal transducer and activator of transcription 3 (STAT3) can downregulate the expression of immune‐stimulating factors such as interferons while increasing the expression of cytokines like IL6 and IL10, thereby exerting significant immune effects.^[^ ^44^ ^]^ We observed that the level of STAT3 phosphorylation in Ccn1‐deficient KPC cells was lower than that in the control group when treated with IFNγ (Figure 5L). Additionally, the mRNA expression levels of Ccl7, Cxcl3, and Csf3 were also significantly decreased when treated with IFNγ (Figure 5M). Similarly, the mRNA level of Il6 in Ccn1‐deficient KPC cells was lower. This difference in Il6 mRNA expression was further accentuated upon treatment with IFNγ (Figure 5N). However, the mRNA levels of Ccl2, Ccl20, Cxcl1, Cxcl5, and Csf2 were not significantly affected by IFNγ treatment (Figure S8E–G, Supporting Information). Notably, the difference in Il6 mRNA level between Ccn1‐deficient KPC cells and the control group was not significantly influenced by cilengitide treatment. Conversely, the difference in Il6 mRNA level between Ccn1‐deficient KPC cells and the control group was rescued when treated with Ccn1 protein and IFNγ compared to treatment with IFNγ alone (Figure S8H, Supporting Information). Thus, our results indicate that KPC cells are unresponsive to IFNγ in the absence of Ccn1.

### 2.6 Ccn1 Deletion Shows Heightened Sensitivity to Immunotherapy and Gemcitabine

**Figure 6:**
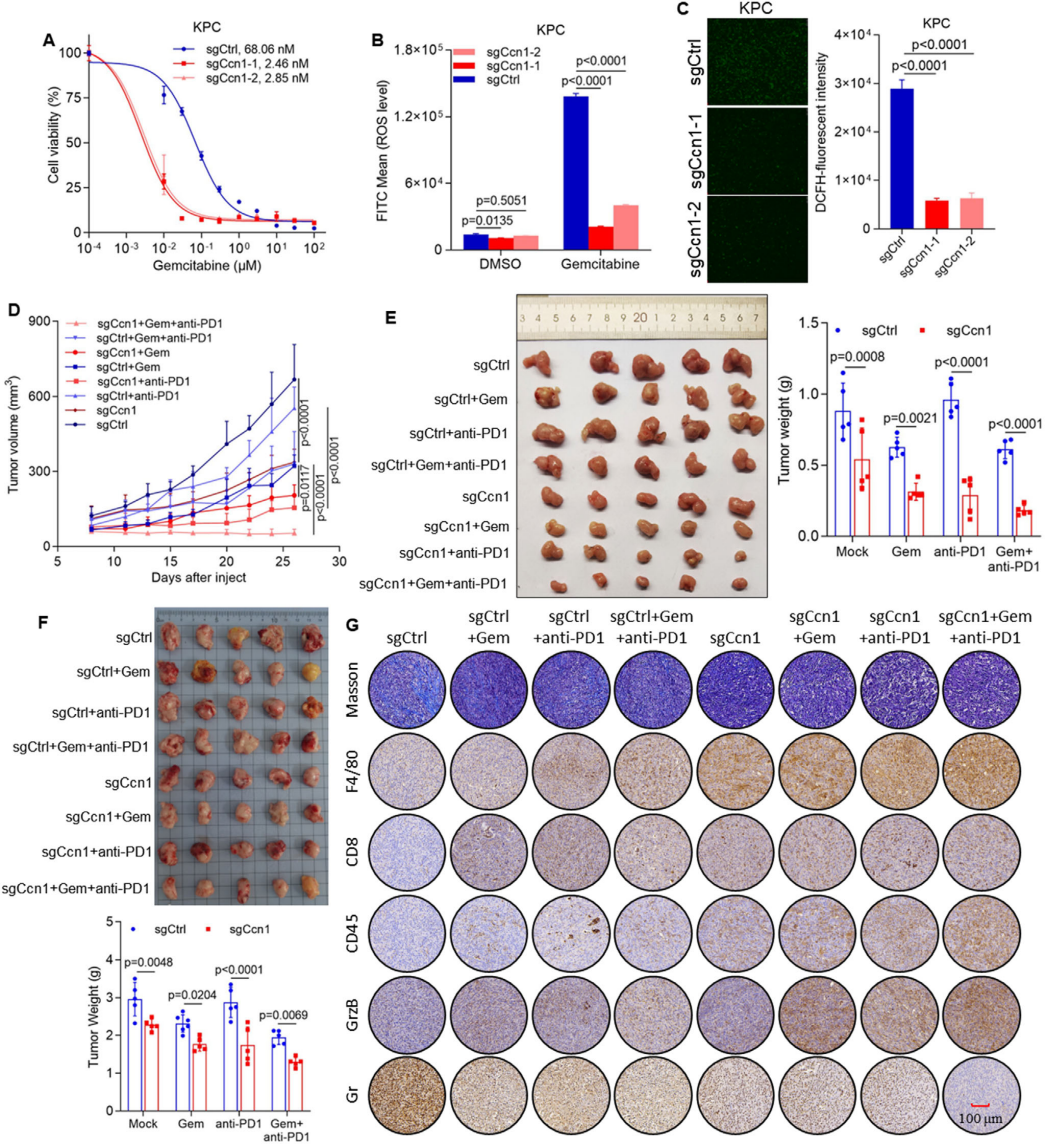
Targeting Ccn1 enhances the efficacy of gemcitabine and anti‐PD‐1 in PDAC. A) Cell viability of sgCtrl and sgCcn1 KPC cells after treatment with indicated concentrations of gemcitabine for 48 h. B) ROS levels in sgCtrl and sgCcn1 KPC cells with gemcitabine for 48 h by Fluorescence‐activated cell sorting (FACS) analysis. C) Representative images (left) and quantification (right) of ROS level in sgCtrl and sgCcn1 KPC cells treated with gemcitabine for 48 h. D,E) C57B/L6 mice were inoculated subcutaneously with sgCtrl and sgCcn1 KPC cells. Tumor‐bearing mice were treated with isotype control, anti‐PD1 mAb, gemcitabine, or a combination of both treatments (*n* = 5 mice per group). Tumor volume was monitored (D), and representative tumor images (left) were acquired with final tumor weights (right) shown (E). F) C57BL/6 mice were orthotopically implanted with sgCtrl or sgCcn1 KPC cells. Tumor‐bearing mice were treated with isotype control, anti‐PD1 monoclonal antibody, gemcitabine, or a combination of both (*n* = 5 mice per group). Representative tumor images (top) and final tumor weights (bottom) are shown. G) Representative images of Masson's trichrome staining and immunohistochemical analysis for F4/80, CD8, CD45, GranzymeB (GrzB), and Gr in sgCtrl and sgCcn1 KPC orthotopic tumors.

For resectable pancreatic tumors, the standard of care involves surgery followed by adjuvant chemotherapy, typically using a combination of gemcitabine plus capecitabine.^[^ ^45^ ^]^ However, chemoresistance is common, posing a significant threat to the long‐term survival of pancreatic cancer patients.^[^ ^46^ ^]^ To investigate whether Ccn1‐deficient KPC cells are sensitive to gemcitabine, we treated these cells alongside control cells with gemcitabine. Intriguingly, Ccn1‐deficient KPC cells displayed increased sensitivity to gemcitabine at lower concentrations (**Figure** **6**A). Moreover, the ROS levels in Ccn1‐deficient KPC cells were significantly lower after treatment with gemcitabine (Figure 6B,C).

Despite the promise of immunotherapy, single‐agent immune checkpoint blockade has shown limited clinical benefits in pancreatic cancer, which remains largely refractory to immunotherapy.^[^ ^3^, ^47^ ^]^ It has been suggested that the CXCR4 pathway modulates the immune microenvironment in pancreatic cancer. The inhibition of the CXCR4‐CXCL12 pathway has been shown to enhance T‐cell access to the TME and increase tumor sensitivity to anti‐PD‐L1 therapy.^[^ ^48^, ^49^, ^50^ ^]^ Ccn1‐deficient KPC cells exhibit decreased chemokine expression and increased immune cell infiltration. To verify whether Ccn1‐deficient KPC cells enhance tumor sensitivity to anti‐PD1 therapy, we subcutaneously injected KPC cells with or without Ccn1 into immunocompetent mice. We initiated intraperitoneal injections of gemcitabine alone, anti‐PD1 antibody alone, or a combination of gemcitabine and anti‐PD1 antibody twice a week, starting on day 8 after tumor inoculation. Our results revealed that while gemcitabine inhibited tumor growth, anti‐PD1 antibody alone did not enhance the inhibition of tumor growth by gemcitabine in the control group. However, Ccn1 deletion enhanced the effectiveness of combination therapy (Figure 6D,E).

To further validate whether Ccn1 deletion can enhances the efficacy of anti‐PD1 and gemcitabine in pancreatic cancer treatment, we established an orthotopic pancreatic tumor model in mice by inoculating KPC cells, followed by treatment with anti‐PD1 and gemcitabine. Consistent with results from the subcutaneous tumor models, gemcitabine alone significantly reduced tumor weight, while anti‐PD1 antibody showed limited effect. However, Ccn1 deletion significantly improved the therapeutic response to both monotherapy and combination treatment (Figure 6F), which revealed increased proportions of CD4^+^ and CD8^+^ cells and macrophages, along with a reduction in MDSCs in Ccn1‐deficient tumors (Figure S9A, Supporting Information). In addition, IHC analysis showed a negative correlation between CCN1 expression and infiltration of CD45^+^, CD8^+^, CD4^+^ T cells, and macrophages, and a positive correlation with MDSC abundance. The proportion of CD45^+^, CD8^+^, CD4^+^ further increased upon gemcitabine or combined gemcitabine and anti‐PD1 treatment (Figure 6G; Figure S9B, Supporting Information).

## 3 Discussion

PDAC is characterized by a dense desmoplastic stroma composed of various components, including cancer‐associated fibroblasts, immune cells, endothelial cells, and neurons.^[^ ^51^ ^]^ This stroma contains abundant ECM components like collagen and hyaluronic acid,^[^ ^32^ ^]^ which act as a barrier, hindering effective drug and immune cell delivery to PDAC cells.^[^ ^10^ ^]^ Targeting PDAC stromal components presents potential therapeutic opportunities, with numerous strategies having been explored over the past decade. Enzymatic targeting of stromal hyaluronic acid has shown promise in inducing antitumor responses in PDAC.^[^ ^52^, ^53^ ^]^ This study sheds light on the critical role of CCN1 in PDAC progression, enabling PDAC cells to resist gemcitabine and anti‐PD1 antibody therapy.

We found that Ccn1‐deficient pancreatic cancer cells exhibit reduced expression of collagens, including Col4a1, Col4a2, Col5a1, Col6a1, Col6a2, Col6a3, and Col8a1. Collagens are active components of the tumor stroma that promote cancer cell proliferation, survival, and metastasis.^[^ ^54^ ^]^ High expression levels of COL1A2, COL2A1, and COL4A1 are associated with poor overall survival in patients.^[^ ^55^ ^]^ Deletion of Col1 homotrimer in cancer cells inhibits tumor progression and enhances T‐cell infiltration.^[^ ^56^ ^]^ However, deletion of Col1 in alpha smooth muscle actin positive (αSMA^+^) myofibroblasts decreases stromal Col1 and accelerates pancreatic cancer progression.^[^ ^36^ ^]^ Our study demonstrates that Ccn1‐deficient PDAC cells exhibit reduced collagen expression and promote the infiltration of CD8^+^ T cells, CD4^+^ T cells, IFNγ^+^ CD8^+^ T cells, and macrophages.

Type IV collagen regulates chemokines CCL5 and CCL7, which promote cancer cell metastasis.^[^ ^57^ ^]^ We identified decreased expression of chemokines in Ccn1‐deficient pancreatic tumor cells. Upon overexpression of Col5a1 in Ccn1‐deficient pancreatic cancer cells, the expression of Cxcl1 and Cxcl5 were upregulated. Depletion of either CXCR2^+^ or CCR2^+^ tumor‐associated macrophages has been shown to augment antitumor immunity.^[^ ^58^ ^]^ Our study demonstrated that tumor inhibition was observed following treatment with CXCR2 and CCR2 inhibitors, whereas the loss of Ccn1 in pancreatic cancer cells does not induce a response. This suggests that Ccn1 regulates collagens and chemokine expression to promote tumor progression.

Tumor‐associated macrophages play a crucial role in regulating immunogenic or immune‐suppressive T cell programming in PDAC. M2 macrophages are known to promote the expansion of Th2 cells and regulatory T cells in PDAC, whereas M1 macrophages recruit Th1 cells and enhance anti‐tumor cytotoxic T lymphocyte activity.^[^ ^59^, ^60^ ^]^ Our study demonstrates that Ccn1‐deficient tumor cells induce greater macrophage infiltration while maintaining the M1 phenotype. Additionally, Ccn1‐deficient tumor cells exhibit sensitivity to TNFα.

IL6, an inflammatory cytokine, is a major contributor to immune evasion in PDAC.^[^ ^61^ ^]^ IL6 activates STAT3 and increases PDAC cell invasion in vitro.^[^ ^62^ ^]^ The inhibition of STAT3 combined with gemcitabine treatment has been shown to improve survival and reduce tumor growth in mice.^[^ ^63^ ^]^ Our findings indicate that Ccn1 regulates Il6 expression and STAT3 activation, and depletion of Ccn1 results in reduced tumor growth. Combining Ccn1 depletion with gemcitabine treatment leads to higher tumor inhibition compared to gemcitabine alone. Interestingly, Ccn1 depletion enhances immunotherapy efficacy, and its combination with gemcitabine demonstrates synergistic effects, suggesting that Ccn1 loss could enhance immunotherapy outcomes.

**Figure 7:**
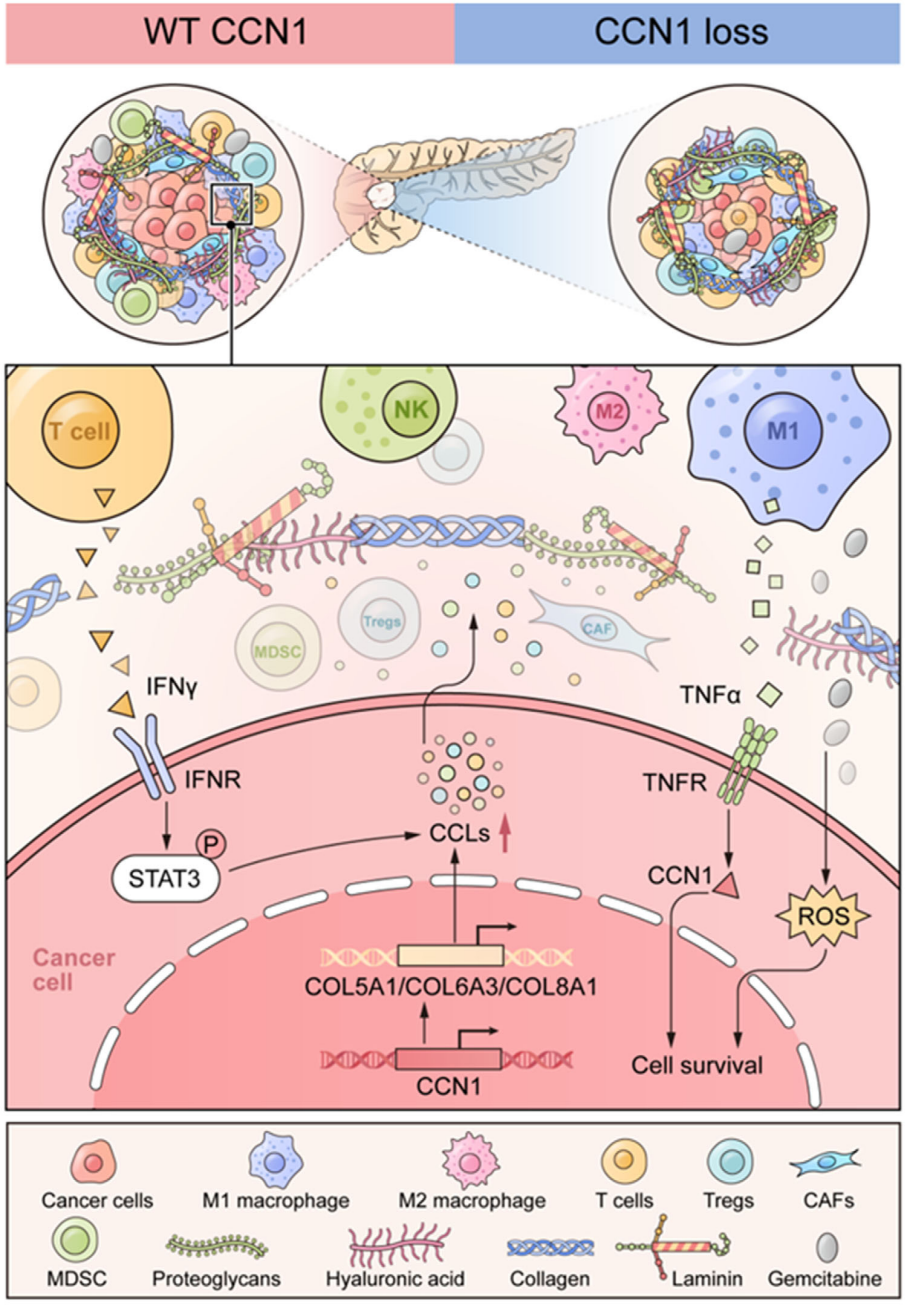
Proposed model for CCN1‐induced tumor immunosuppression in pancreatic cancer. This schematic diagram illustrates the role of CCN1 in pancreatic cancer. In control pancreatic cancer cells, the tumor cells exhibit resistance to TNFα‐mediated cell death. In contrast, CCN1‐ KO pancreatic tumor cells are more sensitive to TNFα‐induced apoptosis. Loss of CCN1 leads to reduced collagen expression, which further downregulates the secretion of chemokines, ultimately promoting immune cell infiltration into the TME.

In summary, our study suggests that Ccn1 repels T cells and recruits MDSCs through Ccn1‐regulated collagens and chemokines (**Figure** **7**). Ccn1 regulates chemokine expression by promoting collagen expression, and chemokine levels remain suppressed in Col6a3‐deficient KPC cells even with IFNγ treatment. Further, pancreatic cancer cells lacking Ccn1 are sensitive to TNFα‐induced cell death and show inhibited polarization from M1 to M2 macrophages. Notably, Ccn1 deletion enhances the efficacy of immunotherapy, effectively inhibiting pancreatic tumor growth. Overall, our findings offer insights into the role of Ccn1 in enhancing the efficacy of both chemotherapy and immunotherapy in PDAC.

## 4 Experimental Section

### Patients and Tissue Samples

PDAC tissues and adjacent normal tissue samples were obtained from patients undergoing surgical procedures at the Affiliated Renmin Hospital of Hubei University of Medicine. All protocols regarding the use of patient samples in this study were approved by the Ethics Committee of the Renmin Hospital (No: 2024KS021). The experiments were conducted in accordance with the Code of Ethics of the World Medical Association and the approved guidelines of the Renmin Hospital. Clinical information details are presented in Table S1 in the Supporting Information.

### Cell Culture

KPC, Pan02, and HEK293T cells were cultured in DMEM (C11995500BT, Gibco) supplemented with 10% FBS (A5669701, Gibco) and 1% penicillin–streptomycin (15140122, Gibco). αVβ3 and αVβ5 integrin inhibitor cilengitide (S6387, Selleck) and DDR1/2 inhibitor VU6015929 (S6817, Selleck) were purchased from Selleckchem. Recombinant mouse CCN1 protein (CSB‐BP006463MO) was obtained from CUSABIO. Mouse TNFα recombinant protein (24095, CST) and mouse IFNγ recombinant protein (39127, CST) were purchased from Cell Signaling Technology.

### Animal Experiments

Specific‐pathogen‐free (SPF) female BALB/c‐Nu/Nu and C57BL/6J mice, aged 6–8 weeks and weighing ≈20 g, were purchased from GemPharmatech Co., Ltd (SCXK(SU)2018‐0008). Upon arrival, the animals were housed in the Laboratory Animal Research Center of Jiangxi Center of Medical Device Testing (SYXK(GAN)2018‐0008) and acclimated to the environment under appropriate feeding conditions for one week. CCR2 inhibitor RS504393 (S9738, Selleck), CXCR2 inhibitor SB225002 (S7651, Selleck), and gemcitabine (S1714, Selleck) were purchased from Selleckchem. In vivo MAb anti‐mouse PD‐1 was purchased from BioXcell (BE0146). All animal experiments were approved by the Institutional Animal Care and Use Committee of Jiangxi Center of Medical Device Testing (Approval number: PZ202201).

All animals were randomized before tumor inoculation. For the subcutaneous model, 1 × 10^6^ or 2 × 10^6^ KPC and Pan02 cells suspended in PBS were subcutaneously injected into the back of each mouse with a total volume of 100 µL. Each condition of mice implanted with tumor cells underwent two treatments, including 200 µg/per anti‐mPD‐1 antibody twice a week, 10 mg/kg gemcitabine twice a week, 10 mg/kg CXCR2 inhibitor SB225002 daily, 5 mg/kg CCR2 inhibitor RS504393 twice a day. Specifically, the anti‐mPD‐1 antibody was administered on days 3, 7, 10, 14, and 17, while gemcitabine was administered daily. Tumor measurements were conducted 3–4 times a week, starting on day 7 after inoculation, until either the survival endpoint was reached, or no palpable tumor remained. Tumor volumes were calculated using the formula for a hemi ellipsoid (volume = length × width × height/2). Mice were sacrificed when tumors reached 20 mm in diameter or 1000 mm^3^ in volume.

For the orthotopic transplantation model, 2 × 10^6^ KPC cells suspended in PBS were mixed 1:1 v/v with Matrigel and slowly injected into the pancreas after mice were anesthetized. Tumor‐bearing mice were then randomly assigned to treatment groups and administered 200 µg/per anti‐mPD‐L1 antibody twice per week and 10 mg/kg gemcitabine twice per week. Mice from the same cohort were euthanized at the endpoint to collect tumor tissues and serum for further analyses.

### Generation of Stable Cell Lines

HEK293T cells were co‐transfected with lentiviral vectors encoding the gene of Ccn1 or sgRNAs, along with packaging vectors psPAX2 and pMD2G, using EZ Trans. After 48 h of transfection, the supernatant containing viral particles was collected and filtered through a sterile 0.45 µm filter. The filtered supernatant was concentrated using Lenti‐X Concentrator and was applied to cell infection for 18 h. Following the infection, the cells were selected using Puromycin (A1113803, ThermoFisher) for one week.

### siRNA Knockdown of Collagens

siRNAs were purchased from Azenta Life Science. To achieve collagen knockdown, we transiently transfected KPC cells with a mixture of siRNAs targeting specific collagens including Col4a1, Col4a2, Col5a1, Col6a1, Col6a2, Col6a3, and Col8a1 using Lipofectamine RNAiMAX (13778150, Invitrogen), following the manufacturer's instructions. After 48 h, levels of collagens and chemokines were measured. Additionally, single siRNAs were individually transfected to specifically knock down each targeted collagen.

### Cell Viability Assay

KPC cells and macrophage were seeded into a 96‐well plate, and cell viability was assessed using the Cell Counting Kit‐8 (CCK‐8) (C0005, TargetMol) assay, following the manufacturer's instructions. KPC cells and macrophage were seeded into a 6‐well plate, and cell viability was assessed using the FACS. The samples were incubated with fluorochrome‐conjugated antibodies against Live/Dead (65‐0865‐14, Invitrogen), CD45 (363‐0451‐82, Invitrogen), CD206 (141720, BioLegend), CD86 (105032, BioLegend), F4/80 (565410, BD) and CD11b (11‐0112‐82, Invitrogen) before analysis by flow cytometry.

### Macrophages Transwell Assay

Transwell assay was conducted using Transwell chambers (3421, Corning). Macrophages were harvested and suspended in a serum‐free medium, adjusting the cell density to 1 × 10^6^ cells per mL. The Transwell insert was filled with 200 µL of the cell suspension, while the lower chamber was filled with 600 µL of 4 × 10^4^ KPC cells. After 24 h, the remaining cells were removed in the upper chamber. The invaded cells were fixed with 4% paraformaldehyde and stained with 0.1% crystal violet. Stained cells were visualized and counted under an inverted microscope.

### Induction of Bone Marrow‐Derived Macrophages (BMDMs)

Bone marrow was harvested from mice, and a single‐cell suspension was prepared by filtering through a 70 µm cell strainer. The isolated bone marrow cells were cultured in RPMI‐1640 medium (C11875500BT, Gibco) supplemented with 10% FBS, 1 × Minimal Essential Medium Non‐Essential Amino Acids (MEM NEAA) Solution (11140050, Gibco), 1% penicillin–streptomycin (15140122, Gibco), 10 × 10^−3^ m HEPES (pH 7.0) (15630080, Gibco), 50 × 10^−6^ m of β‐mercaptoethanol (21985023, Gibco), 1 mM sodium pyruvate (11360070, Gibco), and 20 ng mL^−1^ recombinant mouse macrophage colony‐stimulating factor (M‐CSF) (416‐ML, R&D). After 3 d, nonadherent cells were removed, and the adherent cells were further cultured for an additional three days. To induce the polarization of BMDMs toward the M1 phenotype, cells were treated with 20 ng mL^−1^ IFNγ.

### Flow Cytometry Analysis

Tumors and organs were collected from C57BL/6J mice with pancreatic cancer and immediately placed in ice‐cold PBS. Tumor tissues were mechanically minced using medical scissors and enzymatically digested at 37 °C in DMEM medium containing 2 mg mL^−1^ collagenase type D (11088866001, Roche), 20 units mL^−1^ DNase I (DN25, Sigma), and 200 µg mL^−1^ hyaluronidase (H3506, Sigma) for 30 min. For surface antigen staining, cells were first incubated with mouse CD16/CD32 antibody (14‐0161‐82, Invitrogen) for 10 min at 4 °C, followed by incubation with fluorochrome‐conjugated antibodies against CD45 (17‐0451‐82, Invitrogen), CD3e (561108, BD), CD4 (100545, BioLegend), CD8a (100744, BioLegend), F4/80 (565410, BD), CD11b (11‐0112‐82, Invitrogen), IFNγ (25‐7311‐82, Invitrogen), Granzyme B (12‐8898‐80, Invitrogen), Fixable Viability Dye (65‐0865‐14, Invitrogen) and Ly‐6G/Ly‐6C (Gr‐1) Antibody (108439, BioLegend) for 30 min at 4 °C in the dark. For intracellular antigen staining, cells were fixed with 1X Fixation/Permeabilization solution (00‐5123, Invitrogen) for 30 min at 4 °C and then stained with fluorochrome‐conjugated antibodies in 1X Permeabilization buffer (00‐8333, Invitrogen) for 30 min at room temperature. Cell viability was assessed by staining with the LIVE/DEAD Kit (L34994, Invitrogen). Data acquisition was performed on a Beckman CytoFLEX S flow cytometer, and data analysis was conducted using FlowJo software version 10.

### Macrophage Polarization

Macrophages were isolated from the bone marrow and polarized with 20 ng mL^−1^ IFNγ. After 7 d of stimulation, the polarized macrophages were cocultured with KPC cells. KPC cells were seeded into a 6‐well plate, and the following day, the polarized macrophages were added to the coculture system along with 20 µg mL^−1^ IFNγ. Each condition was performed in triplicate. After 48 h, the tumor cells were washed with PBS and detached from the culture plate using 0.05% trypsin‐EDTA (25200072, Gibco). Subsequently, the samples were incubated with fluorochrome‐conjugated antibodies against CD45 (363‐0451‐82, Invitrogen), CD206 (141720, BioLegend), CD86 (105032, BioLegend), F4/80 (565410, BD) and CD11b (11‐0112‐82, Invitrogen) before analysis by flow cytometry.

### Luminex Multiplex Assay

Cell supernatants and blood samples were collected, and plasma was isolated by centrifugation and stored at −80 °C. Tumor tissues were harvested and homogenized in lysis buffer containing 1 X Protease inhibitor cocktail for Luminex assay (Univ‐bio, Shanghai, China) with. Soluble factors were quantified using the Luminex FLEXMAP 3D multiplexing platform. A mouse Premixed Multi‐Analyte kit (LX‐MultiDTM‐31, LabEx) was used to measure the concentrations of multiple soluble factors, including CCL1, CCL11, CCL12, CCL17, CCL19, CCL2, CCL20, CCL22, CCL24, CCL27, CCL3, CCL4, CCL5, CCL7, CX3CL1, CXCL1, CXCL10, CXCL11, CXCL12, CXCL13, CXCL16, CXCL5, CSF2, IFNγ, IL10, IL16, IL1β, IL2, IL4, and IL6. Luminex assays were performed according to the manufacturer's instructions, with standard curves, quality controls, and background controls included.

### Western Blotting

Cells were lysed in RIPA buffer, and the protein concentrations were determined using the Pierce BCA protein assay kit (23225, ThermoFisher). The cell lysates were boiled and separated on a 10% SDS‐PAGE gel. Proteins were then transferred onto a PVDF membrane (IPVH00010, Millipore), which was incubated with primary antibodies against the following proteins: GAPDH (60004‐1‐Ig, Proteintech), CCN1 (39382S, CST), Caspase‐3 (9662S, CST), c‐Caspase‐3 (9664S, CST), STAT3 (9139S, CST), phospho‐STAT3 (9145S, CST), NF‐κΒ (8242S, CST), phospho‐NF‐κΒ (3033S, CST), COL5A1 (sc‐166155, Santa Cruz) and TNFα (11948, CST). Following incubation with primary antibodies, the membrane was probed with HRP‐conjugated secondary antibodies goat anti‐mouse (SA00001‐1, Proteintech) and goat anti‐rabbit (SA00001‐2, Proteintech), and protein expression was visualized using an ECL kit (34577, ThermoFisher). The membranes were imaged with the ChemiDoc MP Imaging System.

### RT‐qPCR

Total RNA was extracted from cells using the TRIzol Reagent (15596026, Invitrogen), followed by reverse transcription to synthesize the cDNA using PrimeScript RT Reagent Kit (RR047A, Takara). Quantitative real‐time PCR (qRT‐PCR) was performed on the QuantStudio 6 Pro system using a 2X SYBR Green mix (4309155, ThermoFisher). Gene expression data were normalized to the expression levels of GAPDH. Relative gene expression was determined using the 2^−ΔΔCt^ method. Primer sequences are provided in Table S2 in the Supporting Information.

### ELISA Assay

Cancer cells were cultured in DMEM with 10% FBS under indicated conditions. The supernatant was collected and concentrated for detection using ELISA kits (ab253223, Abcam). All procedures were carried out according to the manufacturer's instructions.

### Immunohistochemical (IHC) Staining

Paraffin‐embedded tissues were sectioned into 5 µm slices and stained with hematoxylin and eosin (H&E). For IHC staining, the paraffin slices were dewaxed with xylene, hydrated through a series of gradient alcohols, and permeabilized with 0.1% TritonX‐100 for antigen retrieval. Nonspecific binding was reduced by blocking. The sections were then stained with antibodies against CCN1 (26689‐1‐AP, Proteintech), AACT (RAB‐0011, MXB), AAT (RAB‐0012, MXB), CA199 (MAB‐0778, MXB), CD3 (MAB‐0740, MXB), CD20 (Kit‐0001, MXB), CD34 (Kit‐0004, MXB), CD68 (Kit‐0026, MXB), CD163 (MAB‐0869, MXB), CD3 (MAB‐0740, MXB), Ki‐67 (MAB‐0672, MXB), F4/80 (SC‐377009, Santa Cruz), CD8 (SC‐7970, Santa Cruz), CD3 (SC‐20047, Santa Cruz), CD45 (SC‐1178, Santa Cruz), CD86 (SC‐28347, Santa Cruz), Gr (SC‐393232, Santa Cruz), and GranzymeB (SC‐8022, Santa Cruz) followed by incubation with secondary antibodies (SD3100, Celnovte) for 20 min at 37 °C in the dark. For Masson staining, the paraffin slices were processed according to the manufacturer's instructions (BL1538A, Biosharp). All stained slices were observed and captured under a BX 53 upright microscope (Olympus, Tokyo, Japan).

### Confocal Immunofluorescence Microscopy

Cells were fixed with 4% paraformaldehyde for 30 min, permeabilized with 0.1% Triton X‐100 for 5 min, and blocked with 5% BSA for 1 h. The cells were then incubated with primary antibodies against CCN1 (26689‐1‐AP, Proteintech) overnight at 4 °C. Alexa Fluor 555‐labeled Goat Anti‐Mouse IgG (H+L) secondary antibody (A32727, ThermoFisher) was applied for 1 h at 37 °C, followed by staining with DAPI for 5 min. Images were acquired using an Olympus Confocal Laser Scanning Microscope (Fluoview FV3000).

### Analysis of Ccn1‐Perturbed RNA‐seq Data

To examine the transcriptional changes induced by Ccn1 perturbation, total RNA was extracted from Ccn1‐KO and Ccn1‐wt KPC cells using the TRIzol Reagent (15596026, Invitrogen). The extracted RNA was then subjected to paired‐end mRNA sequencing, conducted by Novogene Co. DEGs were identified based on a fold change threshold of ≥ 2 and a *p*‐value of < 0.05. Subsequent data analysis included heatmap generation and Kyoto Encyclopedia of Genes and Genomes (KEGG) enrichment to highlight significant pathways affected by Ccn1 KO. These analyses were performed using Omicshare bioinformatics tools to uncover pathways potentially involved in PDAC progression regulated by Ccn1.

### Profiling of CCN1 Protein Expression in TCGA Pancreatic Cancer

CCN1 protein expression was quantified in pancreatic cancer samples from TCGA database. The validation of the CCN1 antibodies was confirmed through comparison with immunoblotting.

### Bulk RNA‐seq Analysis

The pancreatic cancer dataset from TCGA‐PAAD^[^ ^64^ ^]^ was utilized for analysis, containing bulk transcriptome data from 182 samples. The transcriptome data of the normal pancreas were obtained from the Genotype‐Tissue Expression (GTEx) database.^[^ ^65^ ^]^ Survival analysis of patients was conducted using the Kaplan‐Meier model with the Python‐based lifelines package.^[^ ^66^ ^]^ Survival differences between the two patient groups were assessed using the log‐rank test.

### scRNA‐seq Analysis

Single‐cell transcriptome data analysis was performed using the Seurat package (version 4.0).^[^ ^67^ ^]^ We analyzed a pancreatic cancer single‐cell transcriptome sequencing dataset containing 1 13 237 cells from 58 patients,^[^ ^68^ ^]^ which includes various annotated cell types such as tumor cells, CD4^+^ T cells, CD8^+^ T cells, fibroblasts, and macrophages. The Seurat package was employed to calculate the average gene expression for each cell type, followed by subsequent correlation analysis.

### Statistical Analysis

All data are presented as mean ± standard deviation (SD). Statistical significance was determined using either one‐way ANOVA, two‐way ANOVA, or Student's *t*‐test with GraphPad Prism 8 software. A *p*‐value < 0.05 was considered statistically significant.

## Conflict of Interest

The authors declare no conflict of interest.

## Author Contributions

H.F. and H.Z. contributed equally to this work. Y.C. and H.F. conceived and designed the study and drafted the manuscript with input from all authors. H.F. performed most of the experiments with assistance from P.Y. and Y.J. H.Z. and L.G. conducted the clinical sample analyses. Y.D. and P.Z. participated contributed to data acquisition and analysis. Y.C., X.L., and Z.C. revised and edited the manuscript. All authors read and approved the final manuscript.

## Supporting information



Supporting Information

## Data Availability

The data that support the findings of this study are available from the corresponding author upon reasonable request.
