# Supplementary material for: CCN1 Enhances Tumor Immunosuppression through Collagen‐Mediated Chemokine Secretion in Pancreatic Cancer
Source: Adv Sci (Weinh). 2025 Apr 27;12(23):2500589. doi: 10.1002/advs.202500589 (PMC12199403; doi:10.1002/advs.202500589)
Supplement: Supplementary file 1 — Supporting Information [file ADVS-12-2500589-s001.docx]

**
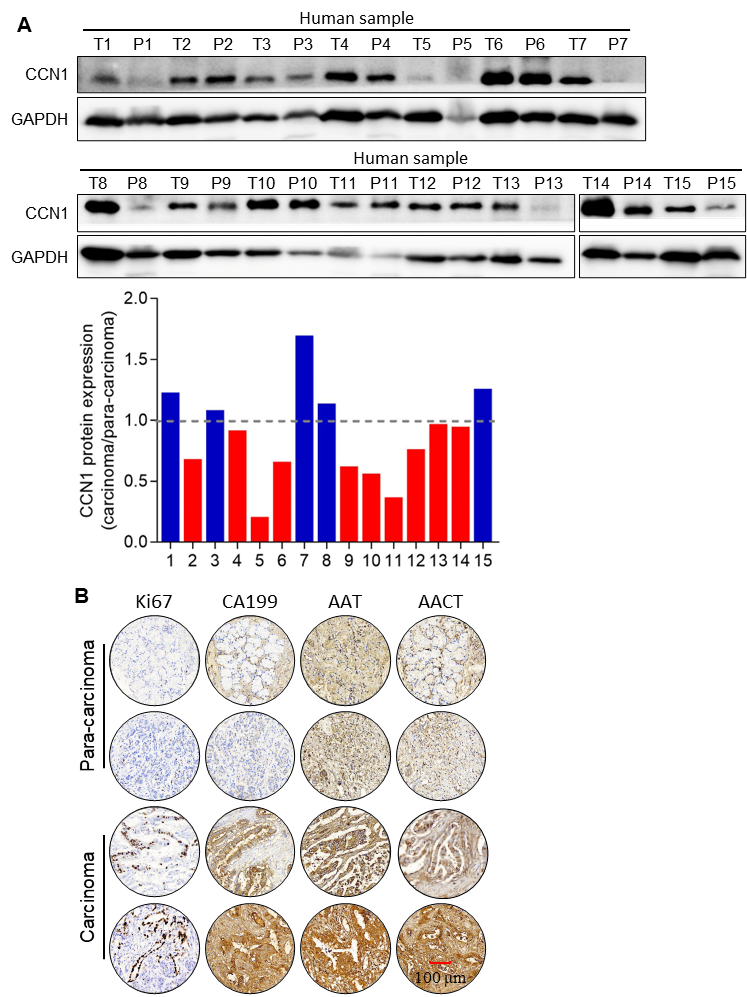
**

**Figure S1.** CCN1 is associated with PDAC. A) Representative CCN1 protein expression (top) and quantification (bottom) in human pancreatic carcinoma tissues (n = 15) and para-carcinoma tissues (n = 15). B) Representative IHC staining comparing Ki67, CA199, AAT, and AACT expression between human pancreatic cancer tissues and normal tissues.


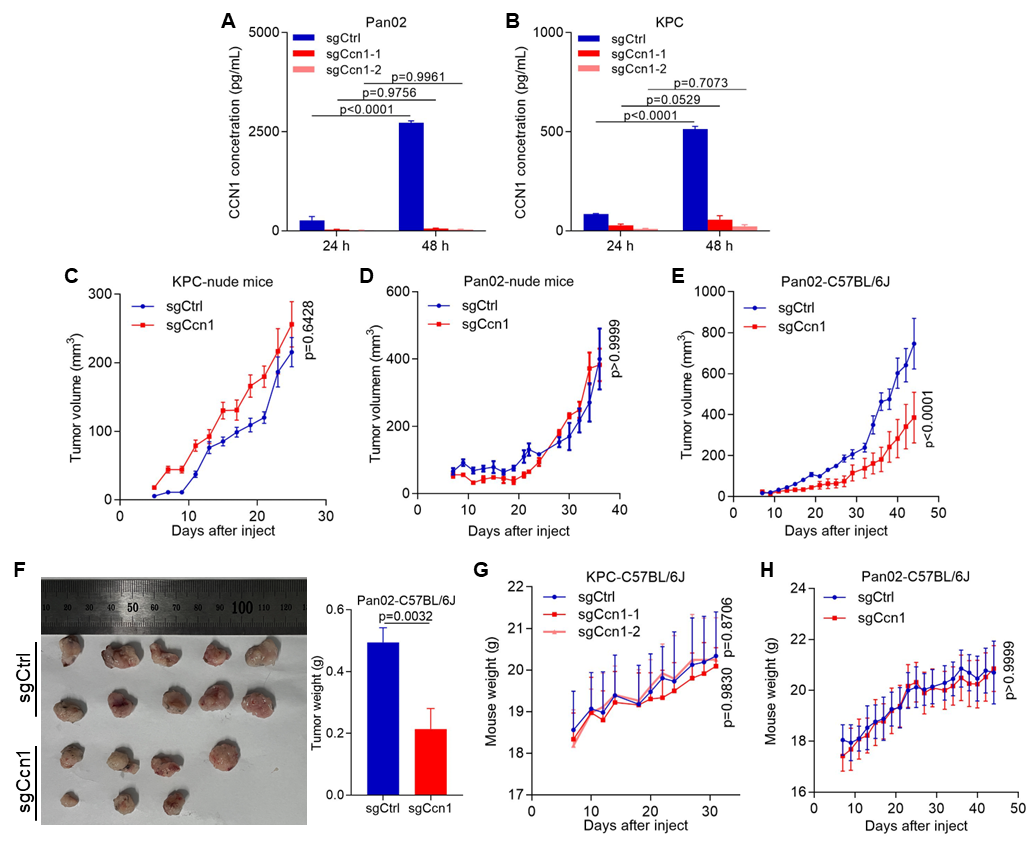


**Figure S2.** Deletion of Ccn1 inhibits tumor growth *in vivo*. A-B) Secreted CCN1 levels in sgCtrl and sgCcn1 KPC cells (A) and Pan02 cells (B). C-D) Tumor growth of sgCtrl and sgCcn1 KPC (n = 5 mice per group) (C) and Pan02 (n = 5 mice per group) (D) cells inoculated subcutaneously into BALB/c-nu mice. E) Tumor growth of sgCtrl and sgCcn1 Pan02 cells inoculated subcutaneously into C57B/L6 mice (n = 7). F) Representative tumor images (left) and quantification bar graphs (right) of sgCtrl and sgCcn1 Pan02 cells. G-H) Mouse weight of sgCtrl and sgCcn1 KPC cells (n = 6 mice per group) (E) and Pan02 (n = 7 mice per group) cells (F).

**
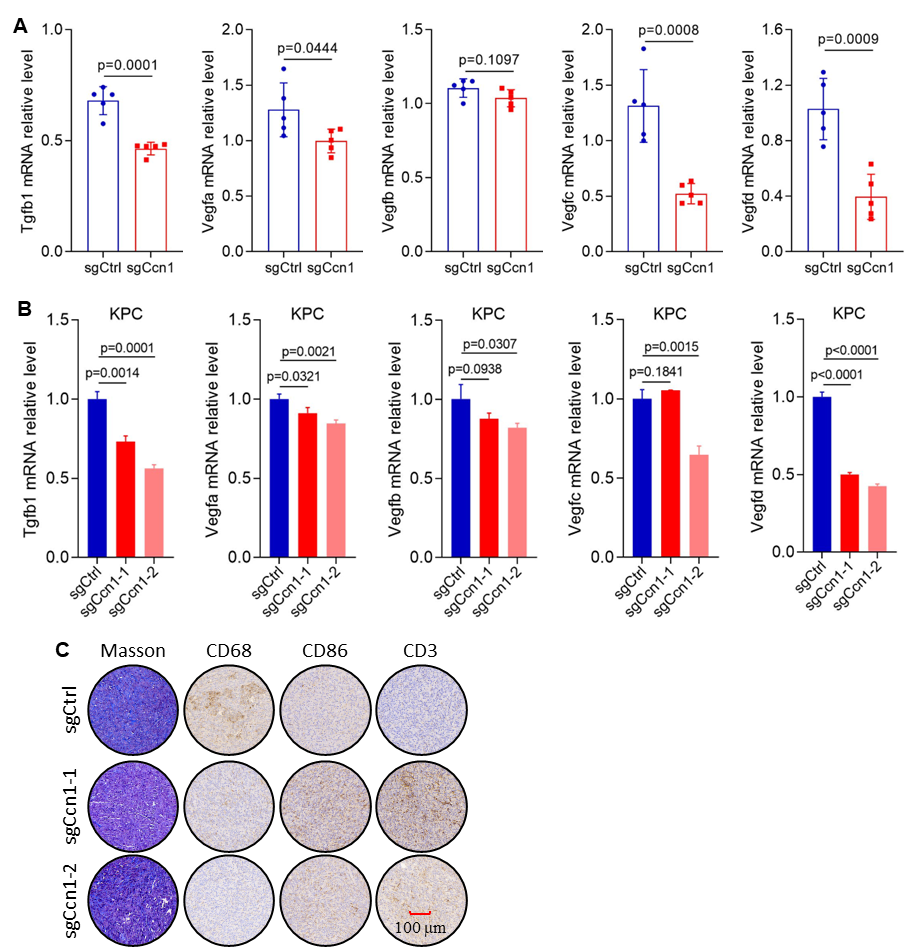
**

**Figure S3.** Ccn1 modulates both VEGF and TGFβ pathways. A) mRNA levels of Tgfb1, Vegfa, Vegfb, Vegfc, and Vegfd in sgCtrl and sgCcn1 KPC tumors. B) mRNA levels of Tgfb1, Vegfa, Vegfb, Vegfc, and Vegfd in sgCtrl and sgCcn1 KPC cells. C) Representative images of Masson’s trichrome staining and immunohistochemical analysis for CD68, CD86, and CD3 in sgCtrl and sgCcn1 KPC tumors.

**
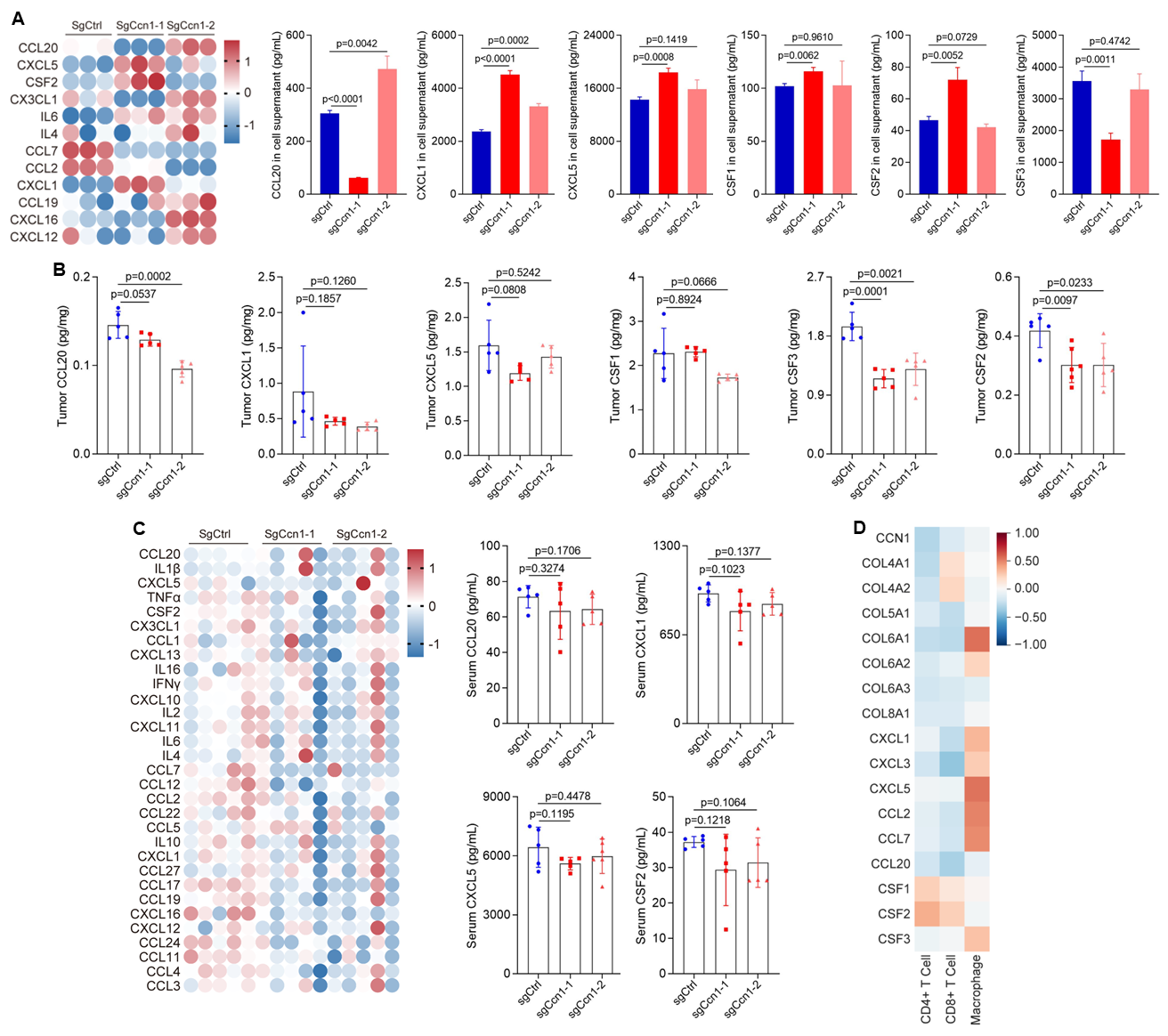
**

**Figure S4.** Ccn1 regulates chemokine and cytokine expression. A) Cytokine levels in the cell supernatant of sgCtrl and sgCcn1 KPC cells, as determined by Luminex multiplex assay. B) Protein levels of CCL20, CXCL1, CXCL5, CSF1, CSF2, and CSF3 in tumors from sgCtrl and sgCcn1 KPC tumors.. C) Cytokine levels in serum from mice bearing sgCtrl and sgCcn1 KPC tumors, as determined by Luminex multiplex assay. D) Heatmap shows the correlation between immune cell proportions and gene expression, including CCN1, collagens, and chemokines, based on analysis of the TCGA-PAAD dataset.


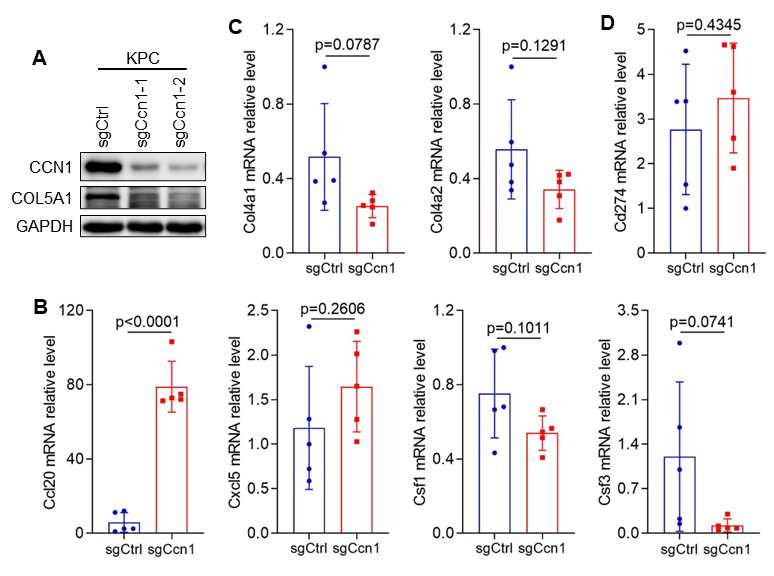


**Figure S5.** Ccn1 regulates the expression of collagens and chemokines. A) Protein expression level of Col5a1 in sgCtrl and sgCcn1 KPC cells. B) mRNA levels of Ccl20, Cxcl5, Csf1, and Csf3 in sgCtrl and sgCcn1 KPC tumors. C) mRNA levels of Col4a1 and Col4a2 in sgCtrl and sgCcn1 KPC tumors. D) mRNA level of Cd274 in sgCtrl and sgCcn1 KPC tumors.


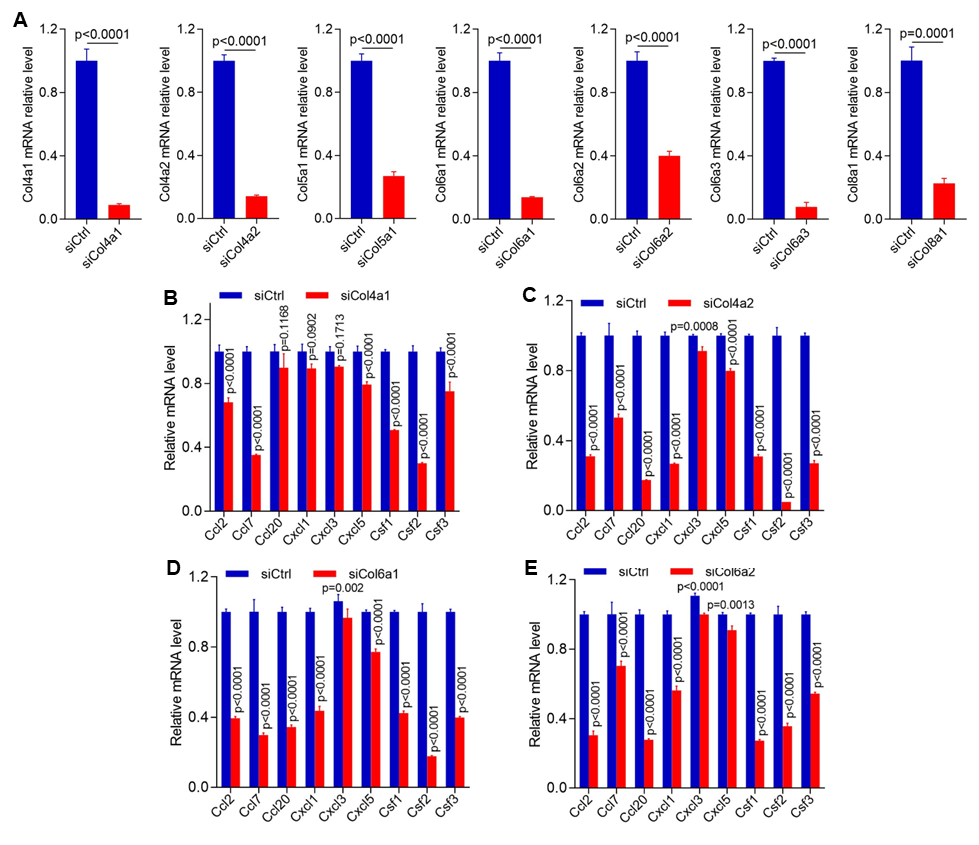


**Figure S6.** Loss of collagens reduces chemokine expression. A) mRNA levels of collagens in KPC cells after the knockdown. B-E) mRNA levels of chemokines, including Ccl2, Ccl7, Ccl20, Cxcl1, Cxcl3, Cxcl5, Csf1, Csf2, and Csf3, in KPC cells after the knockdown of Col4a1 (B), Col4a2 (C), Col6a1 (D), and Col6a2 (E).


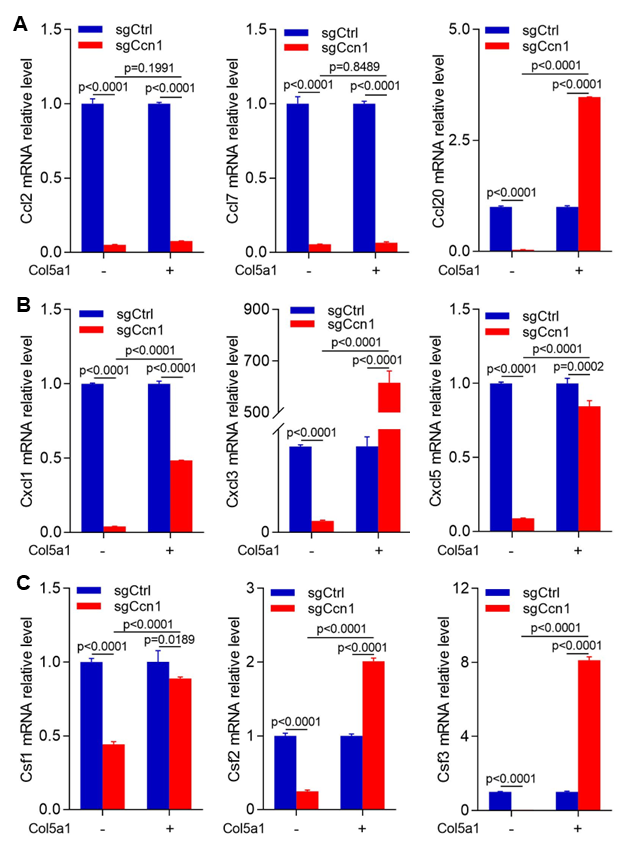


**Figure S7.** Col5a1 overexpression rescues chemokine levels in Ccn1-deficient KPC cells. A-C) mRNA levels of chemokines, including Ccls (A), Cxcls (B), and Csfs (C), in KPC cells after Col5a1 overexpression.


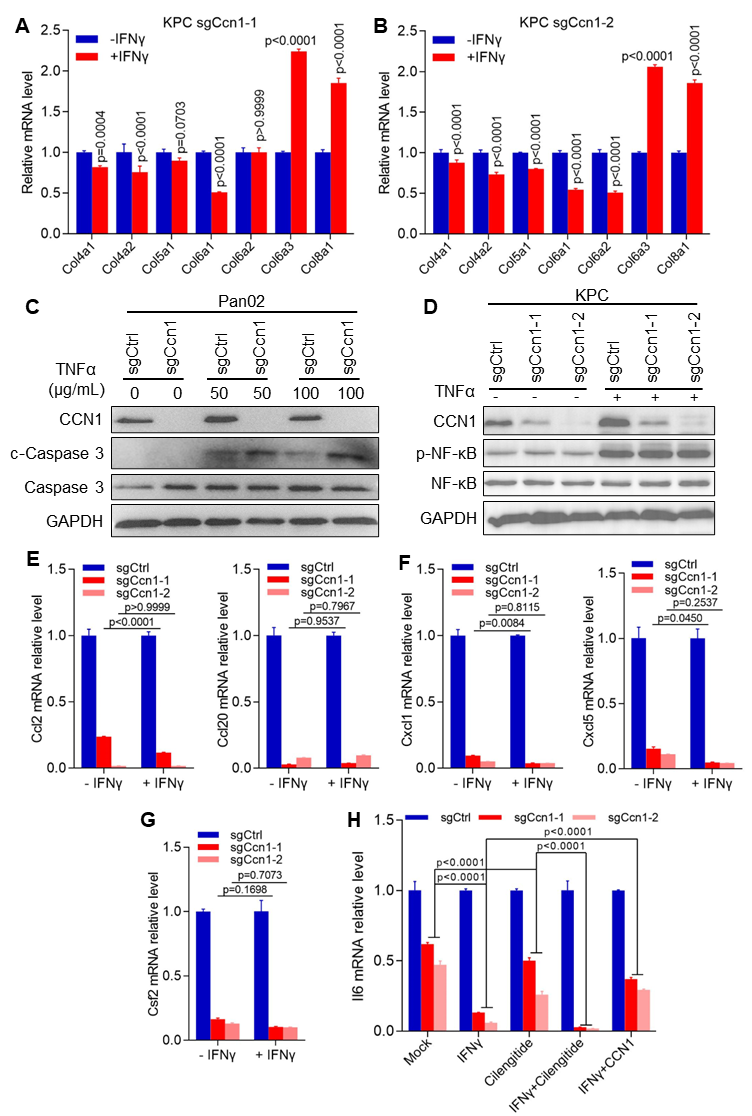


**Figure S8.** Ccn1 regulates chemokine levels in the absence of IFNγ and TNFα. A-B) mRNA levels of collagens, including Col4a1, Cola4a2, Col5a1, Col6a1, Col6a2, Col6a3, and Col8a1 in sgCcn1 KPC cells after treatment with 100 ng/mL IFNγ. C) Protein expression level of c-Caspase 3 in sgCtrl and sgCcn1 Pan02 cells after treatment with TNFα. D) Protein expression level of phosphorylated NF-κB in sgCtrl and sgCcn1 KPC cells after treatment with 100 ng/mL TNFα. E-G) mRNA levels of Ccl2, Ccl20, Cxcl1, Cxcl5, and Csf2 in sgCtrl and sgCcn1 KPC cells after treatment with 100 ng/mL INFγ. H) mRNA level of Il6 in sgCtrl and sgCcn1 KPC cells following treatment with 100 ng/mL IFNγ, 10 μM cilengitide, 200 ng/mL Ccn1 protein, or combination.


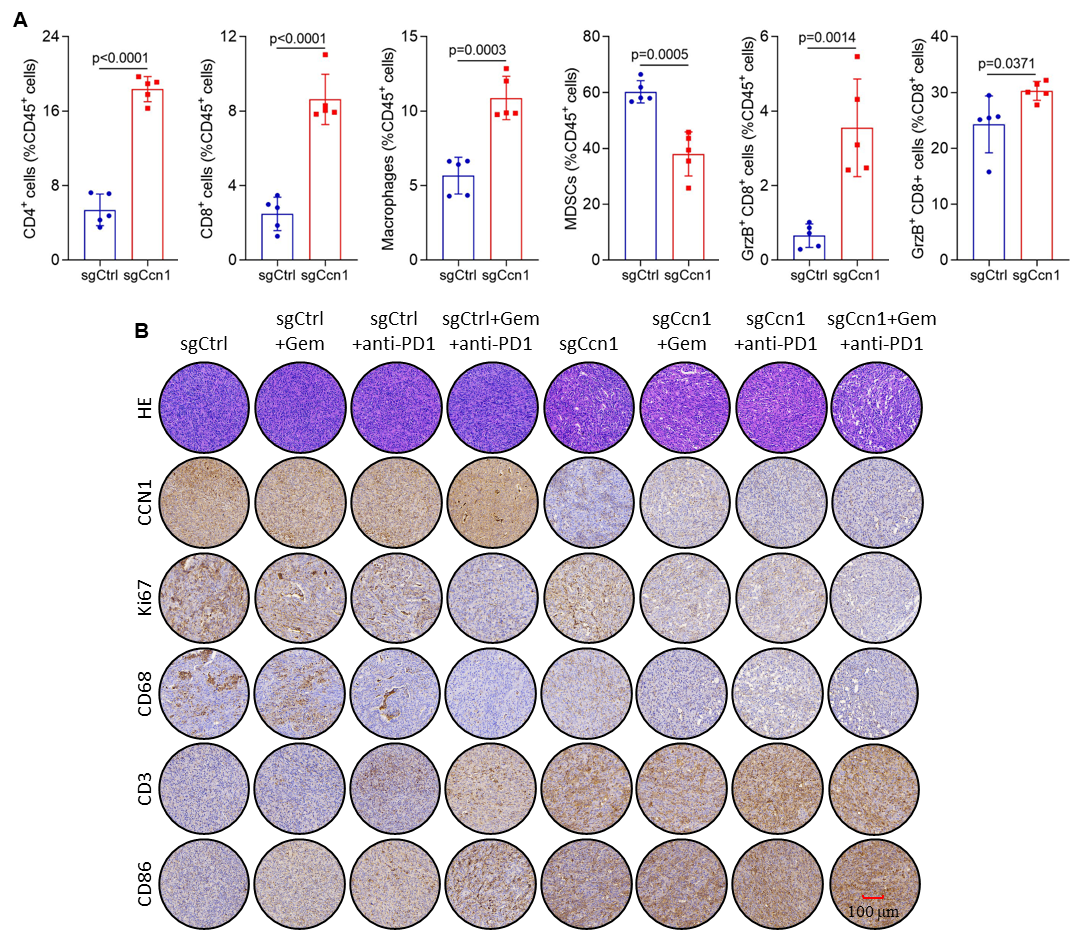


**Figure S9.** Ccn1 inhibits the infiltration of cytotoxic immune cells in pancreatic cancer. A) Flow cytometric quantification of CD4^+^ T cells, CD8^+^ T cells, macrophages, MDSCs, GrzB^+^ and GrzB^+^ CD8^+^ T cells in sgCtrl and sgCcn1 KPC orthotopic tumors. B) Representative images of H&E staining and immunohistochemical analysis for CCN1, Ki67, CD3, CD68, and CD86 in sgCtrl and sgCcn1 KPC orthotopic tumors. Mice were treated with isotype control, anti-PD1 antibody, gemcitabine, or a combination of both.

**Table S1.** Correlation between CCN1 and the clinical characteristics of patients with PDAC.

|  | **Pancreatic ductal adenocarcinoma** | | | | | |
| --- | --- | --- | --- | --- | --- | --- |
| Characteristic | Case  (n = 40) (%) | Negative  (n = 4) (%) | Lower  (n = 8) (%) | Medium  (n = 14) (%) | High  (n = 14) (%) | p value |
| **Gender** |  |  |  |  |  | 0.0387 |
| Male | 28 (70.00) | 2 (7.14) | 6 (21.42) | 10 (35.71) | 10 (35.71) |  |
| Female | 12 (30.00) | 2 (16.67) | 2 (16.67) | 4 (33.33) | 4 (33.33) |  |
| **Age (years)** |  |  |  |  |  | 0.351 |
| <60 | 18 (45.00) | 0 (0.00) | 2 (11.11) | 6 (33.33) | 10 (55.56) |  |
| ≥60 | 22 (55.00) | 4 (18.18) | 6 (27.27) | 8 (36.36) | 8 (18.18) |  |
| **Smoking** |  |  |  |  |  | 0.0387 |
| Yes | 28 (70.00) | 2 (7.14) | 6 (21.42) | 10 (35.71) | 10 (35.71) |  |
| No | 12 (30.00) | 2 (16.67) | 2 (16.67) | 4 (33.33) | 4 (33.33) |  |
| **Grade** |  |  |  |  |  | 0.533 |
| Well | 2 (5.00) | 2 (100.00) | 0 (0.00) | 0 (0.00) | 0 (0.00) |  |
| Moderately | 26 (65.00) | 2 (7.69) | 6 (23.08) | 10 (38.46) | 8 (30.77) |  |
| Poorly | 12 (30.00) | 0 (0.00) | 2 (16.67) | 4 (33.33) | 6 (50.00) |  |
| **Stage (TNM)** |  |  |  |  |  | 0.031 |
| I (IA1-IB) | 26 (65.00) | 4 (15.38) | 6 (23.08) | 8 (30.77) | 8 (30.77) |  |
| II (IIA-IIB) | 6 (15.00) | 0 (0.00) | 0 (0.00) | 2 (33.33) | 4 (66.67) |  |
| III | 8 (20.00) | 0 (0.00) | 2 (25.00) | 4 (50.00) | 2 (25.00) |  |
| **Distant metastasis** |  |  |  |  |  | 0.046 |
| **+** | 10 (25.00) | 0 (0.00) | 2 (20.00) | 4 (40.00) | 4 (40.00) |  |
| **-** | 30 (75.00) | 4 (13.33) | 6 (20.00) | 10 (33.33) | 10 (33.33) |  |
| **Relapse** |  |  |  |  |  | 0.089 |
| **+** | 2 (5.00) | 0 (0.00) | 0 (0.00) | 0 (0.00) | 2 (100) |  |
| **-** | 38 (95.00) | 4 (10.53) | 8 (21.05) | 14 (36.84) | 12 (31.58) |  |
| **Survival state** |  |  |  |  |  | 0.361 |
| Survive | 28 (70.00) | 4 (14.29) | 4 (14.29) | 10 (35.71) | 10 (35.71) |  |
| Die | 4 (10.00) | 0 (0.00) | 4 (100) | 0 (0.00) | 0 (0.00) |  |
| No date | 8 (20.00) | 0 (0.00) | 0 (0.00) | 4 (50.00) | 4 (50.00) |  |

**Table S2.** Primers for qRT-PCR.

| **Gene** | **Forward primer (5' to 3')** | **Reverse primer (3' to 5')** |
| --- | --- | --- |
| Ccn1 | CTGCGCTAAACAACTCAACGA | GCAGATCCCTTTCAGAGCGG |
| Tnfα | CCCTCACACTCAGATCATCTTCT | GCTACGACGTGGGCTACAG |
| Ifng | ATGAACGCTACACACTGCATC | CCATCCTTTTGCCAGTTCCTC |
| Il2 | TGAGCAGGATGGAGAATTACAGG | GTCCAAGTTCATCTTCTAGGCAC |
| Il6 | TAGTCCTTCCTACCCCAATTTCC | TTGGTCCTTAGCCACTCCTTC |
| Il8 | CAAGGCTGGTCCATGCTCC | TGCTATCACTTCCTTTCTGTTGC |
| Tgfb1 | CTCCCGTGGCTTCTAGTGC | GCCTTAGTTTGGACAGGATCTG |
| Vegfa | GCACATAGAGAGAATGAGCTTCC | CTCCGCTCTGAACAAGGCT |
| Vegfb | GCCAGACAGGGTTGCCATAC | GGAGTGGGATGGATGATGTCAG |
| Vegfc | GAGGTCAAGGCTTTTGAAGGC | CTGTCCTGGTATTGAGGGTGG |
| Vegfd | TTGAGCGATCATCCCGGTC | GCGTGAGTCCATACTGGCAAG |
| Col4a1 | CTGGCACAAAAGGGACGAG | ACGTGGCCGAGAATTTCACC |
| Col4a2 | GACCGAGTGCGGTTCAAAG | CGCAGGGCACATCCAACTT |
| Col5a1 | CTTCGCCGCTACTCCTGTTC | CCCTGAGGGCAAATTGTGAAAA |
| Col6a1 | CTGCTGCTACAAGCCTGCT | CCCCATAAGGTTTCAGCCTCA |
| Col6a2 | AAGGCCCCATTGGATTCCC | CTCCCTTCCGACCATCCGAT |
| Col6a3 | GCTGCGGAATCACTTTGTGC | CACCTTGACACCTTTCTGGGT |
| Col8a1 | ACTCTGTCAGACTCATTCAGGC | CAAAGGCATGTGAGGGACTTG |
| Ccl2 | TTAAAAACCTGGATCGGAACCAA | GCATTAGCTTCAGATTTACGGGT |
| Ccl7 | GCTGCTTTCAGCATCCAAGTG | CCAGGGACACCGACTACTG |
| Ccl20 | GCCTCTCGTACATACAGACGC | CCAGTTCTGCTTTGGATCAGC |
| Cxcl1 | CTGGGATTCACCTCAAGAACATC | CAGGGTCAAGGCAAGCCTC |
| Cxcl3 | TGAGACCATCCAGAGCTTGACG | CCTTGGGGGTTGAGGCAAACTT |
| Cxcl5 | GTTCCATCTCGCCATTCATGC | GCGGCTATGACTGAGGAAGG |
| Csf1 | ATGAGCAGGAGTATTGCCAAGG | TCCATTCCCAATCATGTGGCTA |
| Csf2 | GGCCTTGGAAGCATGTAGAGG | GGAGAACTCGTTAGAGACGACTT |
| Csf3 | ATGGCTCAACTTTCTGCCCAG | CTGACAGTGACCAGGGGAAC |
| Cd274 | GCTCCAAAGGACTTGTACGTG | TGATCTGAAGGGCAGCATTTC |
| Ddr2 | ATCACAGCCTCAAGTCAGTGG | TTCAGGTCATCGGGTTGCAC |
| Gapdh | AGGTCGGTGTGAACGGATTTG | TGTAGACCATGTAGTTGAGGTCA |
